# Characterisation of mesenchymal stromal cells in clinical trial reports: analysis of published descriptors

**DOI:** 10.1186/s13287-021-02435-1

**Published:** 2021-06-22

**Authors:** Alison J. Wilson, Emma Rand, Andrew J. Webster, Paul G. Genever

**Affiliations:** 1 Department of Biology, University of York, York, YO10 5DD UK; 2 Science and Technology Studies Unit, Department of Sociology, University of York, York, YO10 5DD UK

**Keywords:** Mesenchymal stem cells, Mesenchymal stromal cells, Clinical trial, Characterisation, Cell therapy, Regenerative medicine

## Abstract

**Background:**

Mesenchymal stem or stromal cells are the most widely used cell therapy to date. They are heterogeneous, with variations in growth potential, differentiation capacity and protein expression profile depending on tissue source and production process. Nomenclature and defining characteristics have been debated for almost 20 years, yet the generic term ‘MSC’ is used to cover a wide range of cellular phenotypes. Against a documented lack of definition of cellular populations used in clinical trials, our study evaluated the extent of characterisation of the cellular population or study drug.

**Methods:**

A literature search of clinical trials involving mesenchymal stem/stromal cells was refined to 84 papers upon application of pre-defined inclusion/exclusion criteria. Data were extracted covering background trial information including location, phase, indication, tissue source and details of clinical cell population characterisation (expression of surface markers, viability, differentiation assays and potency/functionality assays). Descriptive statistics were applied, and tests of association between groups were explored using Fisher’s exact test for count data with simulated p value.

**Results:**

Twenty-eight studies (33.3%) include no characterisation data. Forty-five (53.6%) reported average values per marker for all cell lots used in the trial, and 11 (13.1%) studies included individual values per cell lot. Viability was reported in 57% of studies. Differentiation was discussed: osteogenesis (29% of papers), adipogenesis (27%), and chondrogenesis (20%) and other functional assays arose in 7 papers (8%). The extent of characterisation was not related to the clinical phase of development. Assessment of functionality was very limited and did not always relate to the likely mechanism of action.

**Conclusions:**

The extent of characterisation was poor and variable. Our findings concur with those in other fields including bone marrow aspirate and platelet-rich plasma therapy. We discuss the potential implications of these findings for the use of mesenchymal stem or stromal cells in regenerative medicine, and the importance of characterisation for transparency and comparability of literature.

**Graphical abstract:**

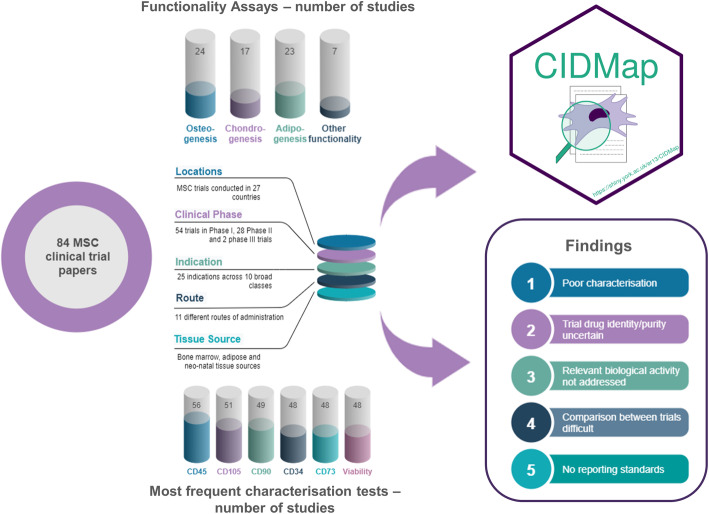

**Supplementary Information:**

The online version contains supplementary material available at 10.1186/s13287-021-02435-1.

## Introduction

Cell-based therapies, often using stem cell populations from adult tissues, offer substantial potential clinical benefits but represent considerable scientific and regulatory challenges in translation [1–3]. Non-haematopoietic stem cells have been identified in the bone marrow, with colony-forming, self-renewal and multi-lineage differentiation capacity demonstrated in vivo [4–7]. These stem cells have acquired a more general identity in the literature, in which in vivo properties have been extrapolated to stromal cells from a wide range of tissues. However, MSC heterogeneity is well established and present at every level of analysis. Compared to their bone marrow counterparts, stromal cells from the umbilical cord, cord blood, adipose, dental pulp, placenta and many other sources, exhibit differing marker profiles, differentiation potential and immunomodulatory properties [8–10]. Clonal populations may differ considerably in their functionality [11–13]. Heterogeneity of morphology and function has been described even within colonies expanded from single cells [14]. Heterogeneous in origin and biological properties, these cells are described by a range of names including mesenchymal stem cell, mesenchymal stromal cell and multipotent progenitor cell; the literature contains many articles discussing identity, stemness and appropriate nomenclature for these most widely studied cells in vitro [15–19]. We do not intend to address the nomenclature issue in this study other than to explore the choice of terms ‘stem’ and ‘stromal’ versus likely mechanisms of action; thus, we adopt the acronym ‘MSC’ throughout without prejudice to the terminology debate.

MSCs have become a cornerstone of cell-based therapy and regenerative medicine, due in no small part to a range of attractive properties including multi-potential differentiation and expression of immunomodulatory and anti-inflammatory molecules in vitro, in vivo and in clinical use [20, 21], although a large-scale clinical success has remained elusive [22, 23]. It is apparent that the use of any cells in regenerative medicine, not least the broad, ill-defined class represented by the term ‘MSC’, requires in-depth characterisation of phenotype, trophic factor expression and potential mechanisms of action [24].

MSCs are reported to be the most frequently studied stem cells in clinical trials [25], with almost 1000 clinical trials registered in the USA alone [26]. The majority of trials are small, uncontrolled studies with differences in design making it challenging to compare and contrast outcomes [27]. A recent analysis examined >1000 stem cell clinical trials, of which 50% were early phase investigations (phases I–II) [28].

The International Society for Stem Cell Research (ISSCR) updated guidelines [29] include the need for standards addressing, amongst other aspects, the reporting of stem cell clinical trials. Analysis of 393 completed stem cell clinical trials against the ISSCR guidelines highlighted the absence of key data including the primary and secondary outcomes and called for the development of guidelines for publication of, in particular, early clinical studies [28]. The existing background literature documents concerns over reporting of cell therapy clinical trials [28, 30, 31], with a lack of clear definition of the trial intervention (study drug) being identified as a significant concern [31–34]. This suggested that analysis of the extent of characterisation parameters being included in papers should be undertaken. Characterisation and standardisation of the cell-based product, combined with the determination of optimum patient characteristics, both to maximise treatment potential and to assist elucidation of mechanisms of action, are key challenges for cell therapy [18, 27, 35]. As clinical development proceeds, more extensive data should become available concerning the safety and efficacy of the product. This published literature should therefore provide a reasonable picture of the overall clinical utility of a product.

Cell-based medicines, unlike other novel biological medicines, may be produced not only by pharmaceutical companies but also in hospitals by research physicians. This is permissible to a limited extent in the EU by an exemption to the requirements of the advanced therapy medicinal products (ATMP) regulation [36] which provides for the manufacture of an ATMP for a specific patient without a marketing authorization, provided the product is manufactured to specific standards of quality and produced on a non-routine basis for use in a hospital within the same member state. In the USA, regulations permit the sale of minimally manipulated human tissues and cells without the Food and Drug Administration (FDA) approval subject to certain conditions [37]. However, the possibility for manufacture outside of the standard medicines paradigms, coupled with the ready supply of dubious miracle cure stories in the media, makes cell-based ATMPs not only a fertile ground for extensive study but has also led to various clinics offering commercial treatments involving unlicensed (unapproved) medicines [38–40]. Unsurprisingly, the safety and efficacy of such unregulated cell-based therapies are of significant concern to regulators [41–43] and the US FDA has recently issued several ‘Warning Letters’ (formal notification that a company is in violation of federal law or regulations) [44, 45]. Concerns have been expressed regarding the rapid progression of MSC-based therapies to the clinic without a clear understanding of the biology underpinning potential mechanisms of action [46–48]. Indeed, the recent Cochrane review of MSC in graft-vs-host disease (GvHD) following haematopoietic stem cell transplantation concluded that evidence was both of low quality and not supportive of MSC efficacy in treating GvHD [49]. The literature covering clinical trials on ATMPs is thus particularly important in conveying the true extent of reliable clinical research in a range of indications, and therefore, the quality of the data published in this regard should withstand scrutiny.

Set against a background of historical concerns over MSC identity and biological activity and calls for a clearer definition of cell therapies in clinical trials, here we have examined trials published in the scientific literature between 2010 and 2019 that used MSCs in a range of clinical indications. We evaluated reporting of the extent of MSC characterisation, defined as information on the expression of cell surface antigens (CD markers), cell viability, differentiation potential and functional assays. The data are made available through “Cell Identity-MSC Application” (CIDMap) (https://shiny.york.ac.uk/er13/CIDMap), an interactive web application which we have developed to allow users to review and perform their own analyses of our dataset. We discuss the potential implications of the findings and make recommendations on how to advance the field based on consistent, defined scientific reporting standards.

## Materials and methods

### Literature review

**Fig. 1:**
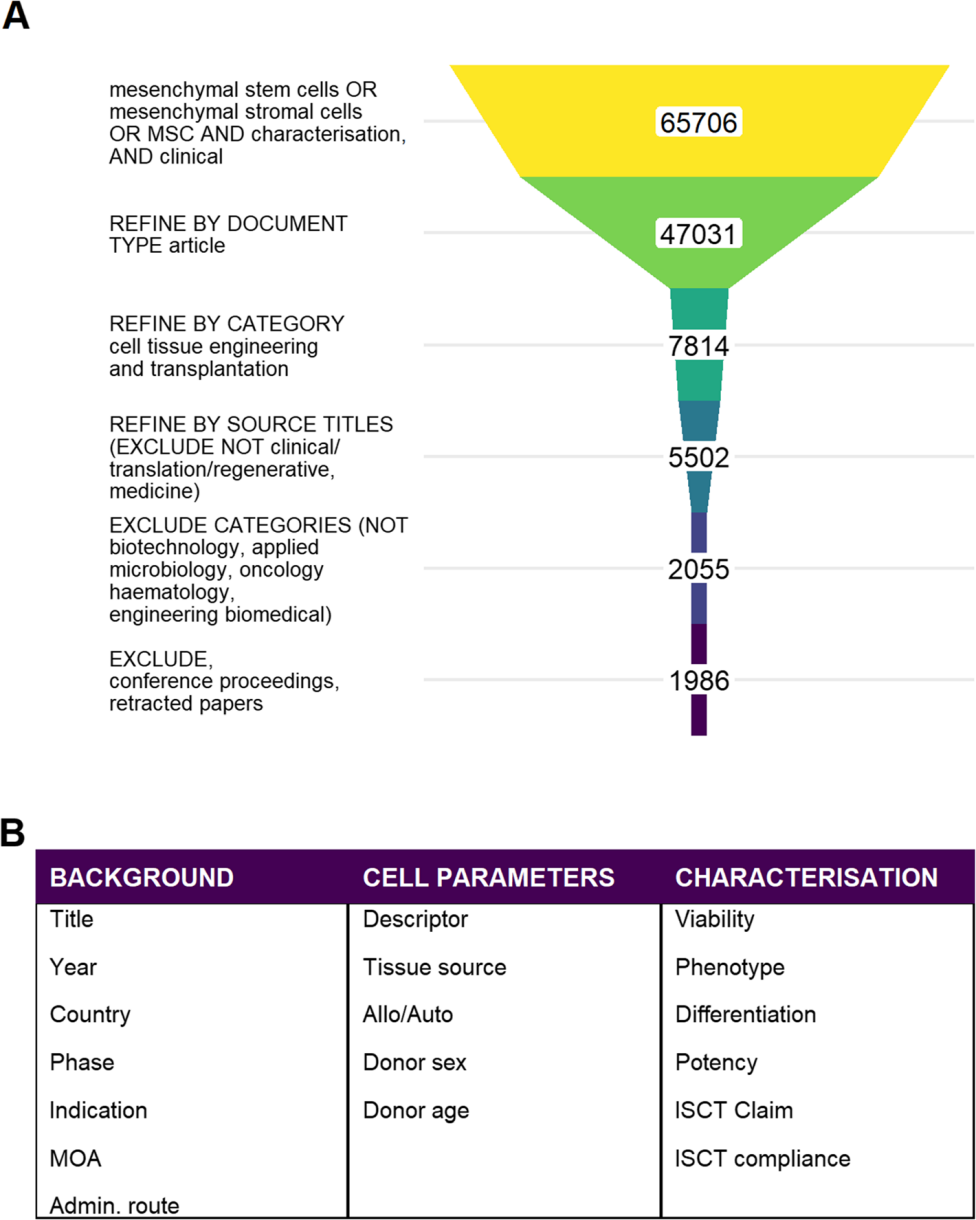
Literature search strategy and results. **A** The schematic shows search terms, refinements and exclusions used. Numbers refer to the total number of papers remaining at each stage. **B** Reported characteristics for MSCs in clinical research studies: data elements captured for this analysis. Basic information on the trial included clinical phase, indication, route of administration and mechanism(s) of action. Specifics of the cell source included donor details, tissue source and usage (allogeneic/autologous) and the descriptor used by the study: stem/stromal cells or other nomenclature. Aspects of characterisation reported in the study were captured, focussing on assessment of viability, phenotypic profile, differentiation capacity and potency evaluations. Reference to ISCT minimal criteria for identification of MSC was also recorded

A literature search of Web of Science was conducted to identify relevant primary clinical research articles based on title and abstract content (Fig. 1A). Application of inclusion/exclusion criteria (Table 1) to the output of the initial search (1986 papers) provided the initial database of papers.

**Table 1:** Inclusion/exclusion criteria

Inclusion	Exclusion
In English	Not in English
MSC or mesenchymal stem cells or mesenchymal stromal cells	Not mesenchymal stem/stromal cells e.g. not stromal vascular fraction, bone marrow aspirate, cord blood, platelet-rich plasma, bone marrow mononuclear cells, induced pluripotent stem cell-derived MSC, conditioned medium
‘Tissue-derived’ stem cells	Not human cells
Human cells	Non-clinical study
Human application (i.e. not non-clinical)	In vitro study only
Clinical application (i.e. not in vitro)	Forward-looking perspective
Research article	Reviews
MSC from any tissue source	Published pre-2010
Characterisation of the population for clinical use	
Published 2010–2019	

In this study, the term ‘characterisation’ was defined as information on the expression of cell surface antigens (cluster of differentiation (CD) markers), cell viability, differentiation potential and functional assays. Data collection tables were designed to capture a range of characteristics and other relevant study parameters. The International Society for Cell and Gene Therapy (ISCT) minimal criteria recommended for defining multipotent mesenchymal stromal cells [50] (expression of CD73, CD90, CD105, absence of CD34, CD45, CD14 or CD11b, CD79α or CD19, HLA-DR expression, plus differentiation in vitro to osteo-, chondro- and adipogenic lineages) were captured. In addition, we noted any mention of expression of a range of other phenotypic markers reportedly typical for MSCs (CD29, CD44, CD146, CD166, CD271, STRO-1, MSCA-1, SSEA-4) or indicative of potential cellular impurities in the MSC population (CD3, CD13, CD31, CD133). The data capture strategy included elements of trial description, cell source and aspects of characterisation (Fig. 1B).

### Definitions

Where the paper identified the clinical trial phase, this was recorded in our analysis. If the stage of clinical development was not defined by the authors, a ‘phase’ designation was entered based on conventional definitions (see Supplementary Information). The phase term was then further condensed into three categories: phase I (first-in-human, safety/initial proof of concept), phase II (exploratory) and phase III (confirmatory) to explore associations between the clinical trial phase and the extent and stringency of characterisation reported.

Mechanism of action ascribed to the MSC within the trial was assigned based on the authors’ own comments and discussion. Where the authors did not clearly state their view, a designation was assigned based on the broad principle theme of mechanism given most prominence or credence by authors (see Supplementary Information). Thus:

- Paracrine = secretion of molecules including mediators of anti-inflammatory or anti-apoptotic effects, host cell recruitment or growth factor expression
- Immune = specifically immunomodulatory effects e.g. in GvHD, transplant tolerance
- Differentiation = in situ differentiation to site-appropriate cell type(s) anticipated
- Multi = multiple relevant mechanisms discussed by authors
- NS = not stated: no discussion, or no clear preference for any of the possible mechanisms of action by which cells were likely to achieve the intended therapeutic effect

The route of administration was recorded using, where possible, the European Directorate for the Quality of Medicines standard terms [51]. Potency/other functionality assays were captured where mentioned, including the expression of relevant proteins, cellular activity assays and differentiation to relevant lineages. This last is distinct from the recording of tri-lineage differentiation as part of routine identification of MSCs.

The extent of cell surface marker characterisation and cell viability reported in the literature set was recorded and articles were categorised as reporting:

1. The percentages of cells which were positive or negative for phenotypic markers for each batch of cells
2. The average percentage of cells which were positive or negative for phenotypic markers across the trial
3. That cells were tested as positive or negative for phenotypic markers but without the percentages
4. The cells were of a ‘standard’ phenotype or referenced published literature
5. No information about phenotypic markers and/or viability

The number of categories was then reduced to allow clearer visualisation of the most commonly reported markers. Reports for which actual values (individual or averaged) were included were combined into a ‘Performed, value reported’ category. Reports for which it was stated that tests had been done, but results were not included, were coded as ‘Performed, value not reported’, and instances in which there was no information in the report relating to testing were combined into a ‘Not mentioned’ category.

### Data analysis

Analysis was conducted in R [52] with tidyverse packages [53] and Microsoft Excel. Descriptive statistics captured numbers of studies by year, by clinical phase, by indication, by route of administration and by putative mechanism of action (MOA). Association between categorical variables was determined with Fisher’s exact tests.

## Results

### Literature search

A literature search of Web of Science was conducted to identify relevant primary clinical research articles based on title and abstract content. Figure 1A illustrates the search strategy and results; Fig. 1B lists the aspects gathered from the papers. Application of inclusion/exclusion criteria (Table 1) to the output of the initial search (1986 papers) provided the initial database of papers.

### Overview of published MSC clinical trials (2010–2019)

A total of 84 papers were included in the analysis. Background information from each trial was summarised including country, clinical phase, indication, route of administration and potential mechanism(s) of action (MOA) of the MSCs (Supplementary Information Table S1).

**Fig. 2:**
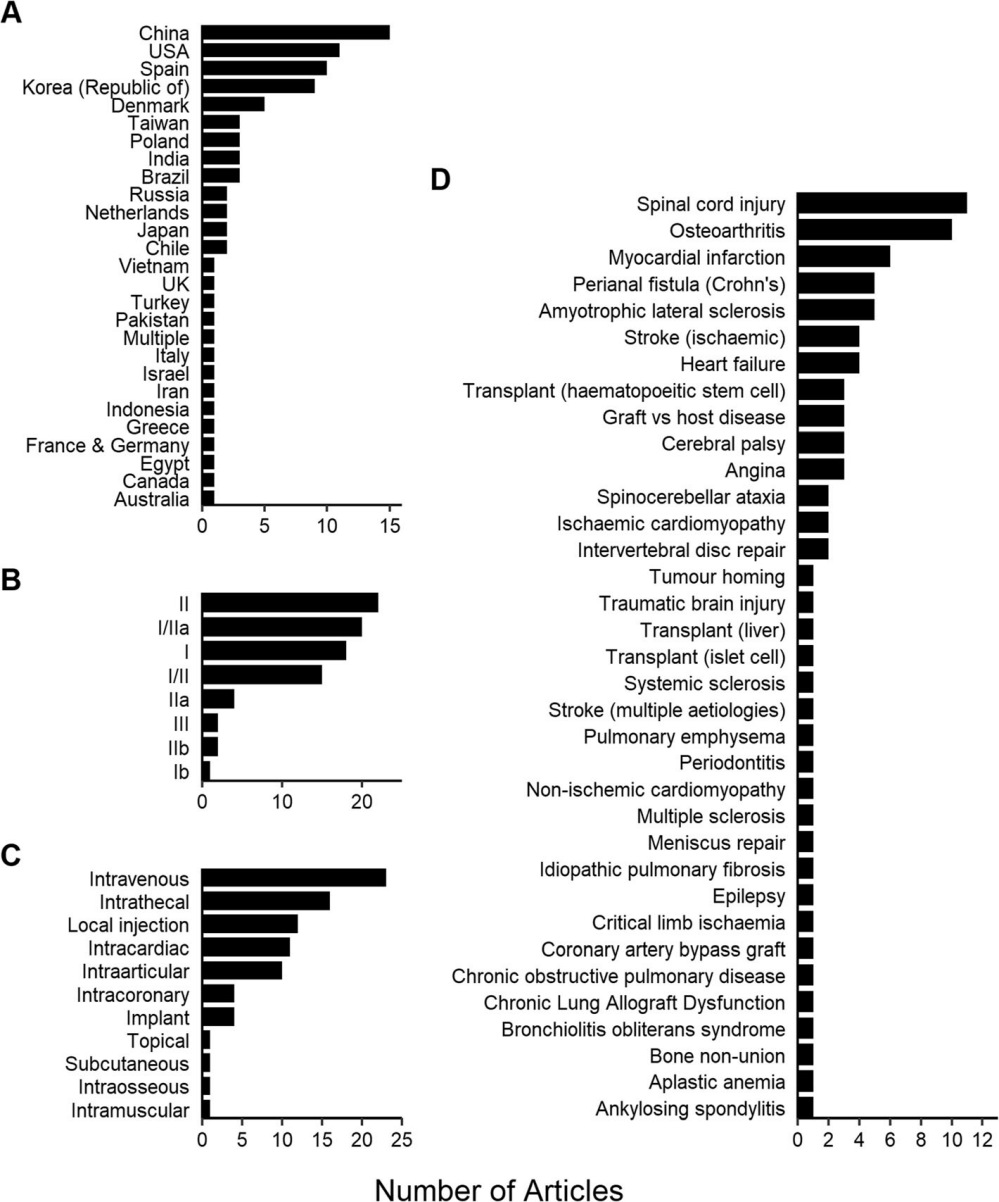
Background trial information. **A** Origin of clinical research publications, ranked by number from each country represented in the analysis. **B** Clinical trials reported in literature by clinical phase, ranked by most commonly represented phase of clinical study. **C** Route of administration, ranked by most commonly used in the studies. **D** Indications addressed by the clinical studies, ranked by most commonly represented indication

MSC-based trials were conducted in 27 different countries. Most studies were conducted in China (15), followed by the USA (11), Spain (10), Republic of Korea (9) and Denmark (5) with between 1 and 4 trials originating from other countries (Fig. 2A). The majority were at early clinical development (safety/proof-of-concept) phase; only two confirmatory (phase III) trials were represented (Fig. 2B). The most frequent routes of administration were intravenous (23), intrathecal (16), local (site-specific) (12), intra-cardiac (11) and intra-articular (10) (Fig. 2C), reflecting the indications being addressed.

The most common indications concerned the nervous system (24) of which 11 studies investigated spinal cord injury repair and five, amyotrophic lateral sclerosis. Cardiovascular indications (16) were broadly spread across myocardial infarction, angina and heart failure. There were 15 reports of musculoskeletal indications of which the majority, 10 studies, concerned osteoarthritis (Fig. 2D).

### MSC tissue sources

**Fig. 3:**
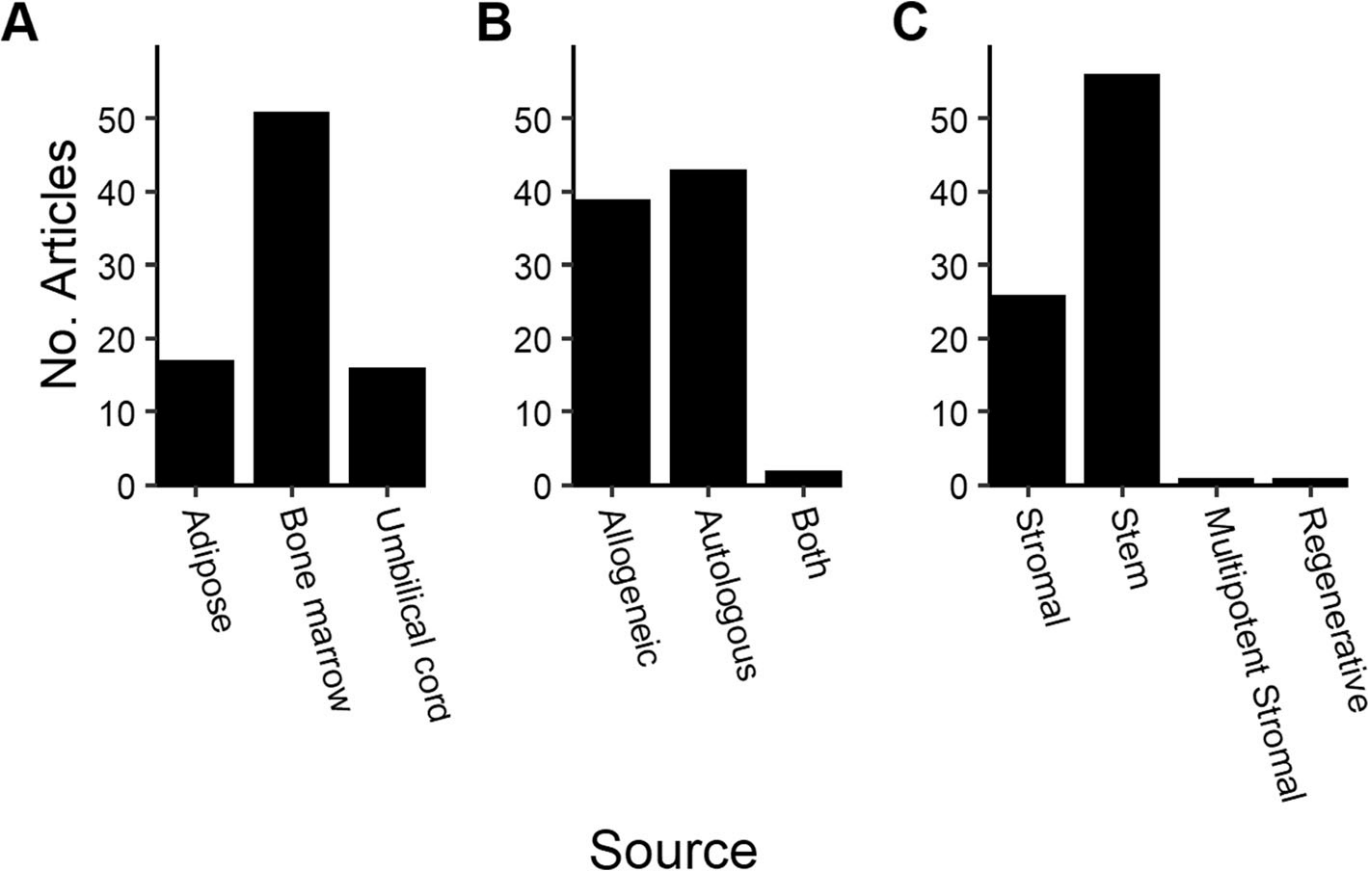
Background information on cells used in clinical trials. **A** Sources of the tissue from which MSCs were derived. **B** Reported use of autologous and allogeneic MSCs. **C** Nomenclature used to describe the cells used in the clinical trials

A range of MSC tissue sources was reported, with the bone marrow representing the most common (51 studies), followed by the adipose tissue (17 studies) and umbilical cord (16 studies) (Fig. 3A). The term ‘umbilical cord’ was used to cover papers reporting the use of MSCs isolated from the umbilical cord blood, umbilical cord and Wharton’s jelly. Autologous cells were used slightly more frequently than allogeneic cells (51% vs 46%), and two papers reported the use of both autologous and allogeneic cells in the same study (Fig. 3B). The term ‘stem’ was much more commonly used than ‘stromal’, with two other individual terms, ‘multipotent stromal’ and ‘regenerative’ cells also being recorded (Fig. 3C).

### MSC characterisation

**Fig. 4:**
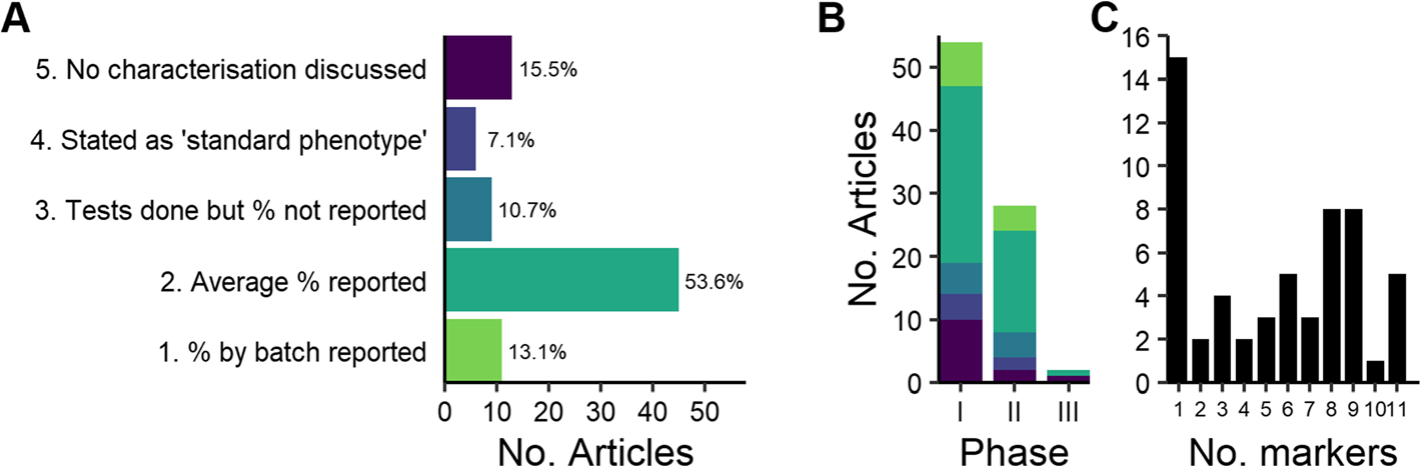
Extent and stringency of characterisation. **A** Number of articles reporting each category of characterisation. **B** Stringency of characterisation reported at each clinical phase of development (coloured as in **A**). **C** Number of phenotypic markers, and viability, evaluated in articles that reported values/averages

Forty-five studies (53.6%) reported the average percentage of cells that were positive or negative for each phenotypic marker tested and/or viability within that trial (‘trial average’). These were presented either as an average for all batches or as a statement that all batches met acceptance criteria (release specification) e.g. ‘all cells met the specification of >90% expression for marker X’. Eleven (13.1%) studies reported the percentages of cells which were positive or negative for phenotypic markers for each batch of product within a trial (‘batch average’). Twenty-eight studies (33.3%) reported no characterisation data. Six of these (7.1%) referred to a ‘standard phenotype’ or other published literature; 9 (10.7%) stated that tests were performed but without reporting values and 13 studies (15.5%) did not discuss any testing, control or evaluation of cells prior to administration to patients (Fig. 4A).

The extent of reporting of CD markers and viability tests performed during studies at each clinical phase was assessed. The most frequent approach was to report average values, generally a single value representing the attribute assessed across the entire clinical population. In each phase of clinical development, there was a large percentage of trials in which no characterisation data were reported: 21/54 (39%) of phase I and 10/28 (40%) of phase II trials (Fig. 4B).

The level of variation in the extent of characterisation between the 56 papers reporting either trial average or batch average values was considerable. The largest subset, 15 papers, included only one characteristic reported by value; in each instance, this was viability. Sixteen (16) papers reported either 8 or 9 characteristics, and the remainder covered fewer characteristics (Fig. 4C). There was no evidence of the association between the clinical trial phase and the extent and stringency of characterisation reported.

**Fig. 5:**
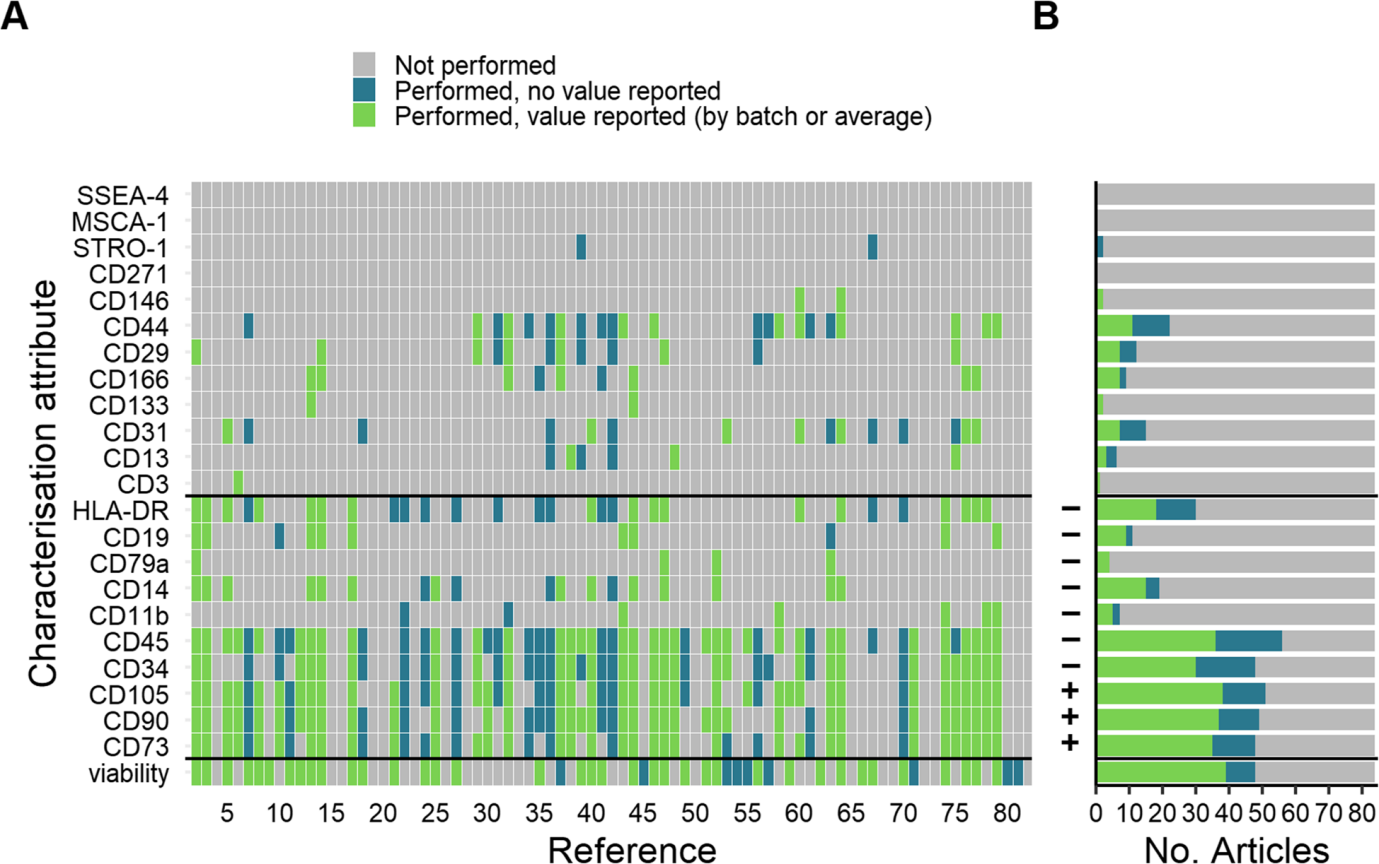
Phenotypic characterisation and viability. The minimal criteria recommended by ISCT for identification of MSC are shown between the black bars on the y-axis. **A** Analysis of individual markers reported in the clinical data set, showing whether an attribute was performed with results reported, whether it was performed but no results stated, or not mentioned in the study report. **B** Number of studies that addressed each attribute, defined by extent of reporting for each marker. Required expression or absence of a marker according to the ISCT recommendation is indicated on the y-axis

For the next part of the analysis, the number of characterisation categories was reduced to three—not performed/performed, no value reported/performed, value reported—to allow clearer visualisation of the most commonly reported markers. The markers/viability assay addressed in each report is shown in Fig. 5A, and the number of reports addressing each marker/viability is shown in Fig. 5B. In four studies viability was the only value reported. Eleven (11) studies reported a value for viability but did not include the values for other characterisation attributes (CD markers) mentioned within the report. Overall, the most commonly evaluated characteristics were a subset of those recommended by ISCT for identification of MSCs: CD45 was assessed in 56 studies, followed by CD105 (51 studies), CD90 (49 studies), CD34 and CD73 (48 studies). One paper documented an analysis of the full set of ISCT markers. Studies that included data on all three aspects (cellular identity, purity and viability) comprised 62% of the dataset. Identity and purity were addressed in 59 studies (70%), and 48 studies (57%) reported measurement of viability prior to administration of the cells to trial subjects.

The surface markers recommended by the ISCT as part of their minimal criteria for identification of multipotent mesenchymal stromal cells are highlighted in Fig. 5. The majority of papers did not report characterisation in line with the ISCT recommendations although 16 papers did mention or specifically claim compliance.

**Fig. 6:**
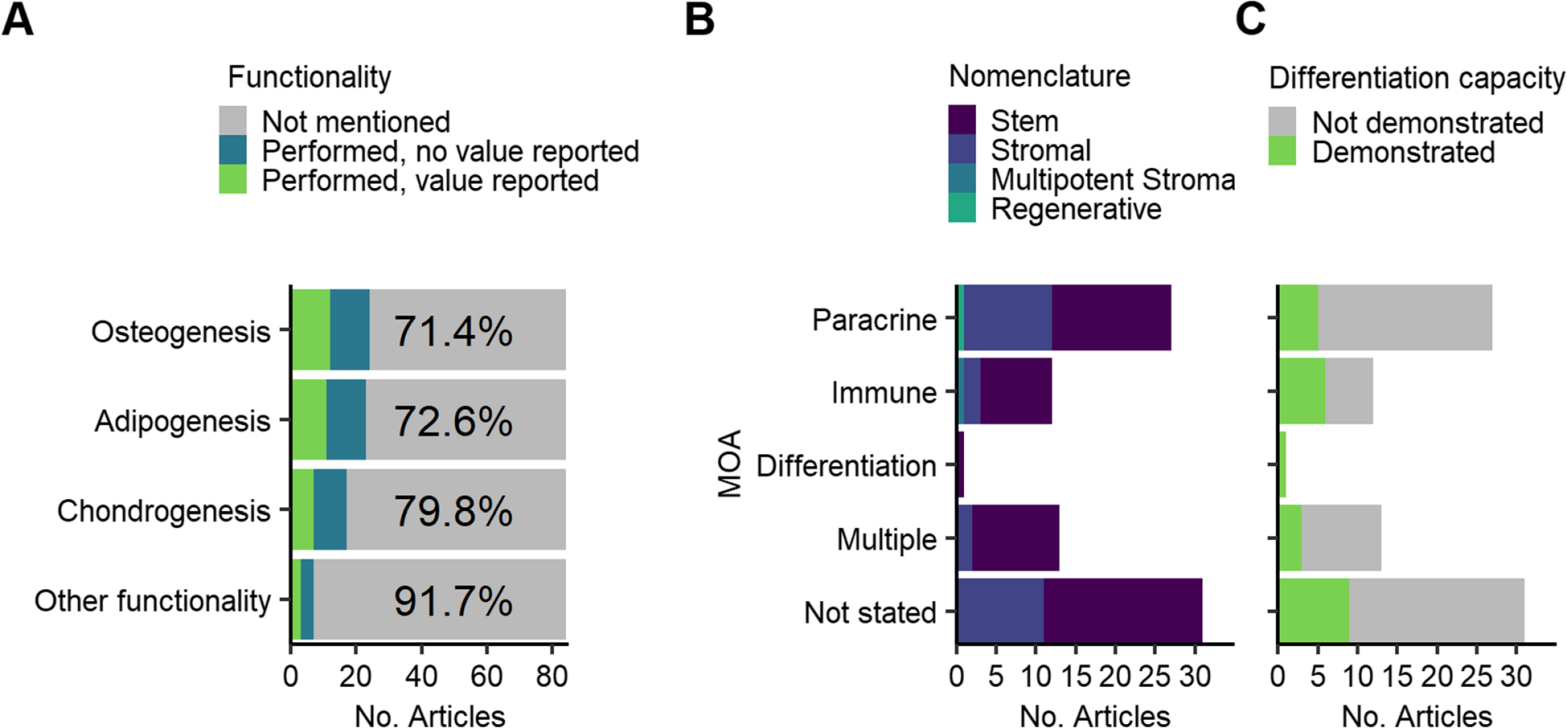
Differentiation and other functionality assessments. **A** Frequency of functionality assessments. **B** Nomenclature (stem/stromal) in relation to potential mechanism of actions relevant to each study indication. **C** Evaluation of MSC differentiation capacity (multi-potentiality) in relation to the mechanism of action anticipated for each study

In vitro differentiation to osteogenic, chondrogenic and adipogenic lineages is an expected property of MSCs: this is a key criterion of the ISCT identification recommendation. Beyond this, the clinical development of medicinal products is required to include the development of one or more potency assays, defined as biological functional attributes relevant to the anticipated clinical mechanism of action of the cells. In the majority of papers, there was no indication that any differentiation potential of the cells had been conducted: osteogenesis and adipogenesis assays were mentioned/discussed in 29% and 27% of studies respectively, chondrogenesis in 20% of papers (Fig. 6A). Functional assessments were identified in 6 papers (7%); these included specific differentiation assays in two papers: one appeared relevant to the intended indication (periodontitis) and one less obviously so (spinocerebellar ataxia). Other functional assays were performed in 4 studies: protein expression in two and assays mentioned but not described in two others. There was no significant association between MOA and the cell description used; mesenchymal ‘stem’ versus ‘stromal’ cell (Fig. 6B) or between MOA and demonstration of differentiation capacity (Fig. 6C).

Papers were examined for claims of compliance with ISCT criteria and the extent to which compliance was actually demonstrated in the paper. Reference was made to standard criteria in 16 papers, of which 10 claimed that the cells used in the study complied with the ISCT criteria (taken to mean both phenotype and multi-lineage differentiation potential). A further 5 papers stated that the cells were consistent with the phenotypic profile alone and one claimed compliance with the phenotype recommended by the ISCT/International Federation for Adipose Therapeutics and Science (IFATS) joint statement for identification of cultured adipose-derived stromal cells (89). However, none of these papers presented data to confirm full compliance of the cells with the standards’ recommendations.

## Discussion

Our analysis has demonstrated that MSC-based clinical trials are being conducted across many countries and for a wide range of indications. The dataset covered 27 countries, 46 specific indications and 11 routes of administration and reported on trials across the spectrum of clinical development stages. Consistent with other analyses [28], we found that the greatest proportion of trial reports covered early trials of safety and initial efficacy (phase I/IIa).

We uncovered a surprising lack of MSC characterisation in published reports. The characterisation is critically important in clinical studies of cell therapies: even with a validated production process, confirmation of the viability and phenotypic identity of the cells being administered to the patient should be the absolute minimum requirement. Assessment of non-target cell types should also be evaluated taking into consideration potential contaminating cells in the source tissue. The extent to which such contaminants may be selected against during the manufacture of the MSC product will vary; thus, evaluation of non-MSC markers should be undertaken as part of quality control, specifically the purity of the clinical cell population. We found that 59 studies (70%) reported some flow cytometric assessment of cell surface markers, most commonly the typically quoted positive expression of CD73, CD90, CD105 and lack of haematopoietic markers CD34 and CD45. Our ranking of reported surface markers by frequency mirrored those in a review of the Investigational New Drug applications submitted to the US FDA [54], reinforcing the idea that despite issues with the ISCT recommendation [48, 55], it has become embedded in the field. Other markers typically used as a positive or negative in MSC populations were reported far less frequently. Three markers suggested in the literature as putative markers for identification and/or selection of MSCS (CD271 [56], MSCA-1 [57] and SSEA-4 [58]) were not adopted in any of the studies we analysed. CD146 [7, 59] and STRO-1 expression were each reported in two studies [60, 61], the latter marker once as a positive identifier of bone marrow-derived cells and once as a negative identifier for expanded adipose-derived MSC.

Considerable heterogeneity of approach was detected amongst papers reporting numerical values for characterisation attributes. The largest subset of studies included average values covering only one characterisation attribute (viability), whereas in the second largest group, 8 studies each reported 8 or 9 attributes, and the remainder covered fewer markers. This suggests that characterisation of the cell population is either undertaken thoroughly or is not seen as a priority. There was no association between the number of characterisation tests reported and the year of publication, suggesting that characterisation, or the reporting of it, is not increasing in importance over time amongst authors.

Only one paper claiming compliance with the surface antigen profile recommended by the ISCT provided data sufficient to confirm this. In 10 papers claiming compliance, the antigen profile reported was not consistent with ISCT: either the marker panel was incomplete or expression values were not consistent with the ISCT recommendation. In the other 5, no data were presented to assess the stated compliance. It should be noted that whilst the ISCT minimal criteria statement for MSCs explicitly confined its application to research, the IFATS/ISCT joint statement on culture-expanded adipose-derived stromal/stem cells [62] was presented as a preliminary tool in the development of standards for clinical use of these cells. It is inappropriate to second-guess the rationale for control of the investigational medicinal product in individual studies, but given that about 17% of studies referred to the ISCT criteria, we may speculate that there is some appetite for reference to an external standard.

Tri-lineage differentiation to osteogenic, chondrogenic and adipogenic lineages in vitro was not demonstrated in 7 of the papers claiming ISCT compliance. In the only paper in which full compliance with the ISCT surface antigen profile was demonstrated, differentiation was not mentioned. The clinical relevance of in vitro differentiation assays, performed or mentioned without data, in 24 studies, was questionable in many instances and may reflect an intention to comply with ISCT recommendations rather than an attempt to confirm biological activity relevant to the indication being investigated. Differentiation assays were conducted in 30% of the studies for indications likely to rely on the secretion of immunoregulatory or anti-inflammatory molecules. Assessment of MSC differentiation capacity would be important for indications based on mechanisms of action involving differentiation. However, there were more studies in which MSC differentiation was demonstrated for an immune MOA, and fewer for paracrine and multiple MOA than expected.

The majority of papers (67%) described the MSC population as mesenchymal *stem* cells, with *stromal* being used in most others (31%), even though stem-related properties were not implied as being relevant for the immunomodulatory and secretome-based indications being investigated. There was no significant association between MOA and nomenclature (stem/stromal).

Distinct from multi-lineage differentiation characterisation of MSCs, only six papers included reference to a potency or functionality assay. The relationship between potency/functional assay and clinical indication in these studies was fairly clear in four cases: thrombospondin expression for osteoarthritis; inhibition of T cell proliferation and cytokine expression in bronchiolitis obliterans syndrome for which immunomodulatory mechanisms were postulated; and osteogenesis for periodontitis and neurotrophic factor secretion in amyotrophic lateral sclerosis. In the remaining two papers, a potency assay was mentioned but there was no information provided concerning the assay performed. Immunoselection of CD271^+^ cells from the initial bone marrow aspirate was anticipated to deliver increased beneficial cytokine and immunomodulatory properties in one study, yet it did not report confirmation that the population administered maintained its high CD271 expression following culture expansion. Although the vast majority of studies were an early phase, evaluating the biological properties of the cells being administered is essential for the field to develop.

A key finding of this analysis is that reporting of characterisation information in MSC therapy clinical trials is poor. Most published reports of clinical trials did not include convincing data on the identity of the MSCs; in other words, the study drug. For small molecules and well-defined biotechnology-derived drug products, this is not an issue: the structure of the drug may be clearly defined by its chemical/biochemical composition and identified to other researchers by a statement of international non-proprietary name or structure. In the case of cell-based ATMPs, the key attributes of the study drug cannot be conveyed by a single term such as ‘mesenchymal stem cell’ due to well-documented difficulties in problems defining this cell type [19, 63, 64] and the impact of tissue source, processing, donor and other factors on expression profile and therefore potentially relevant potency and clinical effect [65]. Whilst we recognise that reference to previous work is a normal part of academic reporting, this is not acceptable for clinical trials on investigational medicinal products: the product being administered to patients is required to be tested or a validated surrogate material in the case of autologous products with limited cell availability. In authorising a clinical trial, regulatory authorities in major jurisdictions do not normally accept data generated from different cell sources, donors, processes or manufacturing sites, nor from previous studies. The field must include much more detail to support the comparison of trials and to provide a clear understanding of exactly what drug substance has been tested.

We found that only 62% of the studies included data on cellular identity, purity and viability. It is recognised that characterisation may have been performed and not included in the publication; indeed, this is very likely given that more extensive data would normally be required to obtain a clinical trial authorization in many jurisdictions including the USA, EU, Japan, Australia and Canada. Increasing depth of characterisation is expected as clinical development proceeds and is considered essential to assess product consistency and process control. Given that characterisation data will have to be generated for clinical trial approvals and in particular for marketing authorisation applications, it could be argued that there is little incentive for clinical trial publications to include any detail of cell populations. Certainly, it may be the case that commercial interests mitigate against such disclosure: this is a relevant consideration in later development and may conflict with intellectual property concerns. For example, enrichment of a specific population based on a particular surface antigen may potentially facilitate increased functional protein expression or differentiation capacity, an interest which a company may not wish to emphasise.

However, we argue that clinical trial publications should include at least basic information on the cell population—the drug substance—being administered, for the following reasons:

1. Researchers should be able to evaluate reports for external validity: the literature on MSCs includes increasing numbers of clinical trial reports that physicians may use to guide treatment decisions. It is therefore reasonable to expect that evidence be provided to demonstrate that the cells are likely to be ‘MSCs’ for comparison purposes.
2. Clinical trial outcomes cannot be assessed in their proper context if the test product has not been defined. The ISCT criteria were not intended to represent release criteria for cells for clinical use and in any case such recommendations do not constitute binding regulatory requirements. In the absence of accepted definitive requirements for clinical ‘MSCs’, studies purporting to use MSCs should include, minimally, evidence of identity, purity and viability of the test population.
3. The community involved in research on clinical application of MSCs must recognise that MSCs are subject to potential misuse on a global scale. The term ‘stealth research’, applied originally to medical start-ups promoting innovative products and solutions without peer-reviewed evidence [66], might also be applied to clinics offering unlicensed cell therapies for a multitude of clinical conditions. Such clinics may not offer peer-reviewed evidence of the validity of their treatments, thereby avoiding scrutiny and engagement with the research community. Reliance on ‘in-house’ (unpublished) data may be suggestive that the technology being promoted is unreliable [67]. Reports with poor definitions of the study drug may be particularly likely to be misrepresented in these circumstances. Importantly, the promotion of unapproved treatments by unregulated clinics may also damage the reputation of the research field and erode public trust in the scientific community when patients are unable to distinguish between properly regulated and controlled therapies from offerings from unregulated clinics [68].

Consideration of the related area of bone marrow aspirate (BMA) therapy illustrates the problem of poor definition in clinical trial reporting. A study by Piuzzi et al. [34] assessing reporting of quantitative data in clinical trials showed that only 30% of the studies gave quantitative details of the composition of the test product, and none of the papers included sufficient detail that another researcher could seek to replicate the production of the BMA preparation. A review of studies of various cellular preparations used in intra-articular injection to the knee, including platelet-rich plasma (PRP), mixed adipose-derived nucleated cells, mixed blood-derived nucleated cells and culture-expanded bone marrow adherent cells [30] identified that whilst the majority reported qualitative surface marker characterisation, only one included a functional assay, and only one study applied the term ‘MSC’ correctly within the context of the ISCT minimal criteria. Similarly, studies on PRP were shown to poorly define preparation protocol or define the study treatment in detail [32].

The need for better reporting of stem cell therapy clinical trials, including standardisation of terminology and nomenclature, better definition of cell sourcing and manufacture, and objective characterisation of cellular populations administered to patients has been highlighted [27, 30–32, 34]. Recognising the issues arising from poor reporting of cell therapy clinical trials, and the need to improve standardisation of reports to facilitate comparisons between trials, an international consensus on a communication of cell therapy studies has been developed [31]. In this document, the use of validated methods (Delphi) to develop a consensus amongst around 40 experts produced a recommendation for a standardised reporting format to describe cell therapies: Donor, Origin of tissue, Separation (production method), Exhibited cell characteristics, Site of delivery (DOSES). The E (exhibited cell characteristics associated with behaviour) attributes recommended for reporting included surface antigen expression, functional or performance attributes and physical attributes of the cell product. Although not focussing specifically on MSCs, these principles should be valuable especially in this most widely used cell type. We strongly endorse the proposal identified in this consensus paper as it proposes a core set of attributes for the reporting of cell therapy studies: donor, tissue origin, manufacture/processing, cellular characteristics and route of administration. Similarly, minimum reporting standards including checklists specific for PRP and MSC-based products have been recommended via Minimum Information for Studies Evaluating Biologics in Orthopaedics (MIBO) [33].

The analysis undertaken here provides a detailed illustration of the lack of published detail in MSC clinical trials, which is highlighted at a general level in the DOSES recommendation. In our analysis, poor definition of the drug substance (phenotypic identity) raises the question of what exactly was administered to the patients, what other cell types (impurities) were given with it and what evidence of biological activity was available. The identity and purity of the MSC population, coupled with cell viability, should be the absolute minimum requirement for the identification of the drug substance under evaluation. Of particular concern is the observation that in 36 studies (43%), there was no mention of viability: this most fundamental parameter was not, apparently, considered to be a sufficiently important attribute or contributor to the effect under evaluation to be reported. Therapeutic efficacy may not require viable cells [69], with some effects of MSCs potentially involving products of dead or apoptotic cells, or phagocytosis by recipient monocytes [70, 71]; however, the viability of any cell preparation would seem to be an essential property to be determined.

Science and medicine journals are increasingly adopting standards to which authors must comply for particular publication types: for example, the Preferred Reporting Items for Systematic Reviews and Meta-Analyses (PRISMA) guidelines for reporting of meta-analyses are now required by 181 journals in the health sciences area [72]. The expectations for reporting of randomised controlled clinical trials (RCT) are addressed by the CONSORT (Consolidated Standards of Reporting Trials) statement [73], first published in 1996 and updated in 2010 [74] which establishes minimum elements of trial design and analysis to be included in RCT reports. The statement includes an explicit requirement for the intervention to be described in sufficient detail to allow another researcher to replicate the study, in particular details of the drug and its administration.

The specific CONSORT provisions for herbal medicines can be considered a model for reporting of cell-based product trials, because of similar difficulties in defining the drug substance. Thus, the CONSORT extension for herbal medicines [75] recommends the inclusion of exact plant species (binomial), part(s) of the plant used, extraction and purification methods and conditions, details of composition and methods of analysis. These recommendations complement, to an extent, the DOSES recommendations and support by analogy the idea of a common required set of data to support the identity of any cell-based product administered during a clinical trial. All three recommendations (DOSES, CONSORT and MIBO) are consistent in promoting a minimal data set to allow for increased transparency and comparability of published reports.

We also examined the publication policy of key journals in the cell therapy field in respect of clinical trial reports and requirements for reporting of cell characterisation. Most expect a checklist for compliance with CONSORT, which specifies information to be included in the report of a clinical trial, and compliance with the International Committee of Medical Journal Editors (ICMJE) policy, a good practice umbrella aimed at all authors, reviewers and publishers of biomedical research. It is notable that we have been unable to locate any specific journal policies regarding minimal datasets for cell therapy clinical trials, when these therapies arguably represent the greatest challenge to clear and transparent identification of study drugs used in human subjects.

The introduction of the CONSORT reporting recommendations for RCT reporting has helped to improve the stringency and completeness of publications in the literature [76, 77]. There are, understandably, concerns around the burden on journal staff of checking compliance, and the possible inadvertent distortion of the literature if non-compliant studies is not submitted for publication [78]. Nevertheless, this should be a secondary consideration to maximising the scientific value of published clinical trials, and therefore, we endorse the principle of minimum reporting content, and the adoption of appropriate guidelines for reporting of cell therapy clinical trials; in particular, a detailed description of the study drug should more adequately reflect the true state of research in this increasingly important area.

We should emphasise that our conclusions are based on published data. It is fully appreciated that trial sponsors will have detailed data held internally and may well have completed additional tests beyond those in their published reports. Scrutiny of available results of clinical trials at https://www.clinicaltrialsregister.eu/ and https://clinicaltrials.gov/ did not reveal any additional characterisation data not published in the papers themselves. Our main objective in reporting this analysis, however, is to highlight the current extent of published characterisation and to suggest that improvements in this regard could have significant benefits to the research community. Given the key role of journals in the dissemination of research, we recommend from our evidence that minimum reporting standards for cell therapy clinical trial reports are universally adopted, perhaps as a further extension analogous to the herbal medicines extension for the CONSORT guidelines.

Our study did not set out to capture clinical trial outcomes, for a number of reasons. We recognised prospectively that analysis of the outcome of a trial would be far more complex than a binary determination of ‘successful/not successful’. Many studies were early phase and outcomes focussed on safety rather than efficacy. Primary endpoints and their assessment criteria often varied across studies for the same indication, and in many papers, the results were reported as a series of observations rather than analysed as an intent-to-treat population. Given that many of the papers reported early phase studies, it was not surprising that some papers did not opine on the success of the treatment but positioned the work as preliminary/feasibility for which follow-up studies would be required. Assessing any correlation between the extent of characterisation and outcome would require accounting for a whole range of clinical variables, including detailed inclusion/exclusion criteria, diagnostic criteria, baseline patient demographics, methods of treatment, clinical monitoring and specific outcomes assessment. The dose of cells would be expected to influence treatment outcomes, but the complexity of measuring this fundamental parameter is highlighted by the lack of characterisation data in itself: even if all studies reported cellular viability (they did not), the inherent assumptions around the homogeneity of this cellular population implies that cell number should relate to clinical effect when it is very likely that only a small subset of administered cells would have the intended activity. A wide range of clinical conditions was included in the study. Some of these indications, such as acute myocardial infarction and spinal cord injury, were represented commonly, whereas for others, e.g. meniscus repair and bronchiolitis obliterans syndrome, only one paper was included in the data set. This, coupled with the complexity of any outcome variable and the number of papers, prevents statistically robust correlations been the degree of characterisation and the trial outcome because the data stratification needed would lead to very small sample sizes.

Adequate disclosure of clinical treatment and transparency regarding preparation and analysis of the investigational drug product should help to improve the overall credibility of the cell therapy field. If there is a higher expectation for peer-reviewed evidence, coupled with transparency and meaningful levels of detail, it should become easier to determine the true balance of evidence for and against the use of particular therapies in specific indications. Thus, the results of our study on MSC clinical trials support and exemplify the need for standardised minimum reporting requirements for cell therapy clinical trials.

## Conclusions

Overall, this study highlights the apparent paucity of characterisation data in MSC clinical trial reports. The extent of characterisation being performed does not appear to be increasing over time, and our data suggest a considerable variation in approach towards the necessity of characterising cell populations. Much greater consideration of potential mechanisms of actions should be expected for publication of trials beyond an initial feasibility and safety (phase I) study. Our study findings are consistent with several recent recommendations for improvement in characterising cell therapy populations generally and exemplify the need for better reporting in respect of MSCs, which are so widely used in many indications. We recommend the adoption of minimal standards of cell population identification and testing to be required in published reports of MSC clinical trials.

## Supplementary Information

**Additional file 1: Table S1**. Clinical Trial Summary Information.

## Data Availability

The data set supporting the conclusions of this article is available for analysis and download at https://shiny.york.ac.uk/er13/CIDMap.

## Abbreviations

ATMP: Advanced therapy medicinal product
BMA: Bone marrow aspirate
CD: Cluster of differentiation
CIDMap: Cell identity-MSC application
CONSORT: Consolidated Standards of Reporting Trials (statement)
DOSES: Donor, Origin of tissue, Separation (production method), Exhibited cell characteristics, Site of delivery
GvHD: Graft-vs-host disease
FDA: Food and Drug Administration (USA)
ICMJE: International Committee of Medical Journal Editors
IFATS: International Federation for Adipose Therapeutics and Science
ISCT: International Society for Cell and Gene Therapy
ISSCR: International Society for Stem Cell Research
MIBO: Minimum Information for Studies Evaluating Biologics in Orthopaedics
MOA: Mechanism of action
MSC: Mesenchymal stem/stromal cell
PRISMA: Preferred Reporting Items for Systematic Reviews and Meta-Analyses
PRP: Platelet-rich plasma
RCT: Randomised clinical trial

## References

[1] Galipeau J, Sensébé L (2018). Mesenchymal stromal cells: clinical challenges and therapeutic opportunities. Cell Stem Cell.

[2] Rousseau CF, Mačiulaitis R, Śladowski D, Narayanan G. Cell and gene therapies: European view on challenges in translation and how to address them. Front Med (Lausanne). 2018;5(158):1–6. 10.3389/fmed.2018.00158 PMC5992383 29911104

[3] Lukomska B (2019). Challenges and controversies in human mesenchymal stem cell therapy. Stem Cells International.

[4] Ding L, Saunders TL, Enikolopov G, Morrison SJ (2012). Endothelial and perivascular cells maintain haematopoietic stem cells. Nature.

[5] Zhou BO, Yue R, Murphy MM, Peyer JG, Sean J (2014). Morrison, Leptin-receptor-expressing mesenchymal stromal cells represent the main source of bone formed by adult bone marrow. Cell Stem Cell.

[6] Méndez-Ferrer S, Michurina TV, Ferraro F, Mazloom AR, MacArthur BD, Lira SA, Scadden DT, Ma’ayan A, Enikolopov GN, Frenette PS (2010). Mesenchymal and haematopoietic stem cells form a unique bone marrow niche. Nature.

[7] Sacchetti B, Funari A, Michienzi S, di Cesare S, Piersanti S, Saggio I, Tagliafico E, Ferrari S, Robey PG, Riminucci M, Bianco P (2007). Self-renewing osteoprogenitors in bone marrow sinusoids can organize a hematopoietic microenvironment. Cell.

[8] Hass R, Kasper C, Böhm S, Jacobs R (2011). Different populations and sources of human mesenchymal stem cells (MSC): a comparison of adult and neonatal tissue-derived MSC. Cell Commun Signal.

[9] Kern S, Eichler H, Stoeve J, Klüter H, Bieback K (2009). Comparative analysis of mesenchymal stem cells from bone marrow, umbilical cord blood, or adipose tissue. Stem Cells.

[10] Wegmeyer H, Bröske AM, Leddin M, Kuentzer K, Nisslbeck AK, Hupfeld J, Wiechmann K, Kuhlen J, von Schwerin C, Stein C, Knothe S, Funk J, Huss R, Neubauer M (2013). Mesenchymal stromal cell characteristics vary depending on their origin. Stem Cells Dev.

[11] James S, Fox J, Afsari F, Lee J, Clough S, Knight C, Ashmore J, Ashton P, Preham O, Hoogduijn M, Ponzoni RDAR, Hancock Y, Coles M, Genever P (2015). Multiparameter analysis of human bone marrow stromal cells identifies distinct immunomodulatory and differentiation-competent subtypes. Stem Cell Rep.

[12] Martínez-Peinado P, Pascual-García S, Roche E, Sempere-Ortells JM (2018). Differences of clonogenic mesenchymal stem cells on immunomodulation of lymphocyte subsets. J Immunol Res.

[13] Russell KC, Phinney DG, Lacey MR, Barrilleaux BL, Meyertholen KE, O'Connor KC (2010). In vitro high-capacity assay to quantify the clonal heterogeneity in trilineage potential of mesenchymal stem cells reveals a complex hierarchy of lineage commitment. Stem Cells.

[14] Rennerfeldt DA, Van Vliet KJ (2016). Concise review: when colonies are not clones: evidence and implications of intracolony heterogeneity in mesenchymal stem cells. Stem Cells.

[15] Caplan AI (2010). What’s in a name?. Tissue Eng Part A.

[16] Caplan AI (2017). Mesenchymal stem cells: time to change the name!. STEM CELLS Transl Med.

[17] Prockop DJ (2007). “Stemness” does not explain the repair of many tissues by mesenchymal stem/multipotent stromal cells (MSCs). Clin Pharmacol Ther.

[18] Sipp D, Robey P, Turner L (2018). Clear up this stem-cell mess. Nature.

[19] Wilson A, Webster A, Genever P (2019). Nomenclature and heterogeneity: consequences for the use of mesenchymal stem cells in regenerative medicine. Regen Med.

[20] Abumaree M, Al Jumah M, Pace RA, Kalionis B (2012). Immunosuppressive properties of mesenchymal stem cells. Stem Cell Rev.

[21] da Silva Meirelles L, Fontes AM, Covas DT, Caplan AI (2009). Mechanisms involved in the therapeutic properties of mesenchymal stem cells. Cytokine Growth Factor Rev.

[22] Mastrolia I, Foppiani EM, Murgia A, Candini O, Samarelli AV, Grisendi G, Veronesi E, Horwitz EM, Dominici M (2019). Challenges in clinical development of mesenchymal stromal/stem cells: concise review. Stem Cells Transl Med.

[23] Caplan H, Olson SD, Kumar A, George M, Prabhakara KS, Wenzel P, et al. Mesenchymal stromal cell therapeutic delivery: translational challenges to clinical application. Front Immunol. 2019;10. 10.3389/fimmu.2019.01645. 10.3389/fimmu.2019.01645 PMC6685059 31417542

[24] Samsonraj RM, Raghunath M, Nurcombe V, Hui JH, van Wijnen AJ, Cool SM (2017). Concise review: multifaceted characterization of human mesenchymal stem cells for use in regenerative medicine. Stem Cells Transl Med.

[25] Teixeira FG, Salgado AJ (2020). Mesenchymal stem cells secretome: current trends and future challenges. Neural Regen Res.

[26] Pittenger MF (2019). Mesenchymal stem cell perspective: cell biology to clinical progress. NPJ Regen Med.

[27] Martin I, Galipeau J, Kessler C, Le Blanc K, Dazzi F (2019). Challenges for mesenchymal stromal cell therapies. Sci Transl Med.

[28] Fung M, Yuan Y, Atkins H, Shi Q, Bubela T (2017). Responsible translation of stem cell research: an assessment of clinical trial registration and publications. Stem Cell Rep.

[29] Daley G (2016). Setting global standards for stem cell research and clinical translation: the 2016 ISSCR guidelines. Stem Cell Rep.

[30] Chahla J, Piuzzi NS, Mitchell JJ, Dean CS, Pascual-Garrido C, LaPrade RF, Muschler GF (2016). Intra-articular cellular therapy for osteoarthritis and focal cartilage defects of the knee: a systematic review of the literature and study quality analysis. J Bone Joint Surg Am.

[31] Murray IR, Chahla J, Safran MR, Krych AJ, Saris DBF, Caplan AI, LaPrade R, Cell Therapies Communication Expert Group (2019). International expert consensus on a cell therapy communication tool: DOSES. JBJS.

[32] Chahla J, Cinque ME, Piuzzi NS, Mannava S, Geeslin AG, Murray IR, Dornan GJ, Muschler GF, LaPrade RF (2017). A call for standardization in platelet-rich plasma preparation protocols and composition reporting: a systematic review of the clinical orthopaedic literature. J Bone Joint Surg Am.

[33] Murray IR, Geeslin AG, Goudie EB, Petrigliano FA, LaPrade RF (2017). Minimum Information for Studies Evaluating Biologics in Orthopaedics (MIBO): platelet-rich plasma and mesenchymal stem cells. J Bone Joint Surg Am.

[34] Piuzzi NS, Hussain ZB, Chahla J, Cinque ME, Moatshe G, Mantripragada VP, Muschler GF, LaPrade RF (2018). Variability in the preparation, reporting, and use of bone marrow aspirate concentrate in musculoskeletal disorders: a systematic review of the clinical orthopaedic literature. J Bone Joint Surg Am.

[35] Fitzsimmons REB, Mazurek MS, Soos A, Simmons CA (2018). Mesenchymal stromal/stem cells in regenerative medicine and tissue engineering. Stem Cells International.

[36] Eudralex. 2007. http://eur-lex.europa.eu/LexUriServ/LexUriServ.do?uri=OJ:L:2007:324:0121:0137:en:PDF. Accessed 5 Oct 2020.

[37] FDA, in 21CFR1271.10, U. D. o. H. a. H. Services, Ed. (https://www.accessdata.fda.gov/scripts/cdrh/cfdocs/cfCFR/CFRSearch.cfm?fr=1271.10). Accessed 7 Oct 2020.

[38] Knoepfler PS, Turner LG (2018). The FDA and the US direct-to-consumer marketplace for stem cell interventions: a temporal analysis. Regen Med.

[39] Lysaght T, Lipworth W, Hendl T, Kerridge I, Lee TL, Munsie M, Waldby C, Stewart C (2017). The deadly business of an unregulated global stem cell industry. J Med Ethics.

[40] Bianco P, Barker R, Brüstle O, Cattaneo E, Clevers H, Daley GQ, de Luca M, Goldstein L, Lindvall O, Mummery C, Robey PG, Sattler de Sousa e Brito C, Smith A (2013). Regulation of stem cell therapies under attack in Europe: for whom the bell tolls. EMBO J.

[41] Marks P, Gottlieb S (2018). Balancing safety and innovation for cell-based regenerative medicine. N Engl J Med.

[42] C. A. T. S. S (2010). Committee for Advanced Therapies and, Use of unregulated stem-cell based medicinal products. Lancet.

[43] FDA. 2020. https://www.fda.gov/vaccines-blood-biologics/consumers-biologics/consumer-alert-regenerative-medicine-products-including-stem-cells-and-exosomes?utm_campaign=What%27sNew2020-07-22&utm_medium=email&utm_source=Eloqua Accessed 29 Oct 2020

[44] FDA. 2019. https://www.fda.gov/inspections-compliance-enforcement-and-criminal-investigations/warning-letters/liveyon-labs-inc-588399-12052019. Accessed 29 Oct 2020

[45] FDA. 2020; Vol. 2020. https://www.fda.gov/inspections-compliance-enforcement-and-criminal-investigations/warning-letters/invitrx-therapeutics-inc-581182-03162020. Accessed 29 Oct 2020

[46] Bianco P, Cao X, Frenette PS, Mao JJ, Robey PG, Simmons PJ, Wang CY (2013). The meaning, the sense and the significance: translating the science of mesenchymal stem cells into medicine. Nat Med.

[47] P. Bianco, Mesenchymal” stem cells. Annu Rev Cell Dev Biol 30, 677-704 (2014), 1, doi: 10.1146/annurev-cellbio-100913-013132. 10.1146/annurev-cellbio-100913-013132 25150008

[48] Robey PG. “Mesenchymal stem cells”: fact or fiction, and implications in their therapeutic use [version 1; peer review: 2 approved]. F1000Research. 2017;6(F1000 Faculty Rev):524. 10.12688/f1000research.10955.1. 10.12688/f1000research.10955.1 PMC5399967 28491279

[49] Fisher SA, Cutler A, Doree C, Brunskill SJ, Stanworth SJ, Navarrete C, et al. Mesenchymal stromal cells as treatment or prophylaxis for acute or chronic graft-versus-host disease in haematopoietic stem cell transplant (HSCT) recipients with a haematological condition. Cochrane Database Syst Rev. 2019. 10.1002/14651858.CD009768.pub2. 10.1002/14651858.CD009768.pub2 PMC6353308 30697701

[50] Dominici M, le Blanc K, Mueller I, Slaper-Cortenbach I, Marini F, Krause D, Deans R, Keating A, Prockop Dj, Horwitz E (2006). Minimal criteria for defining multipotent mesenchymal stromal cells. The International Society for Cellular Therapy position statement. Cytotherapy.

[51] EDQM. Council of Europe. 2020. https://standardterms.edqm.eu/. Accessed 02 Oct 2020.

[52] R Core Team. R: A language and environment for statistical computing. Vienna: R Foundation for Statistical computing; 2021. https://www.R-project.org/.

[53] Wickham et al. Welcome to the tidyverse. J Open Sour Softw. 2019;4(43);1686. 10.21105/joss.01686.

[54] Mendicino M, Bailey AM, Wonnacott K, Puri RK, Bauer SR (2014). MSC-based product characterization for clinical trials: an FDA perspective. Cell Stem Cell.

[55] Barry F, et al. Mesenchymal stem cells: characterization, therapeutic evaluation and manufacturing. Eur Cells Mater. 2008;16(3):2.

[56] Mifune Y, Matsumoto T, Murasawa S, Kawamoto A, Kuroda R, Shoji T, Kuroda T, Fukui T, Kawakami Y, Kurosaka M, Asahara T (2013). Therapeutic superiority for cartilage repair by CD271-positive marrow stromal cell transplantation. Cell Transplant.

[57] Battula VL, Treml S, Bareiss PM, Gieseke F, Roelofs H, de Zwart P, Muller I, Schewe B, Skutella T, Fibbe WE, Kanz L, Buhring HJ (2009). Isolation of functionally distinct mesenchymal stem cell subsets using antibodies against CD56, CD271, and mesenchymal stem cell antigen-1. Haematologica.

[58] Gang EJ, Bosnakovski D, Figueiredo CA, Visser JW, Perlingeiro RCR (2006). SSEA-4 identifies mesenchymal stem cells from bone marrow. Blood.

[59] Harkness L, Zaher W, Ditzel N, Isa A, Kassem M (2016). CD146/MCAM defines functionality of human bone marrow stromal stem cell populations. Stem Cell Res Ther.

[60] Cho YB, Lee WY, Park KJ, Kim M, Yoo HW, Yu CS (2013). Autologous adipose tissue-derived stem cells for the treatment of Crohn’s fistula: a phase I clinical study. Cell Transplant.

[61] Perin EC, Borow KM, Silva GV, DeMaria AN, Marroquin OC, Huang PP, Traverse JH, Krum H, Skerrett D, Zheng Y, Willerson JT, Itescu S, Henry TD (2015). A phase II dose-escalation study of allogeneic mesenchymal precursor cells in patients with ischemic or nonischemic heart failure. Circ Res.

[62] Bourin P, Bunnell BA, Casteilla L, Dominici M, Katz AJ, March KL, Redl H, Rubin JP, Yoshimura K, Gimble JM (2013). Stromal cells from the adipose tissue-derived stromal vascular fraction and culture expanded adipose tissue-derived stromal/stem cells: a joint statement of the International Federation for Adipose Therapeutics and Science (IFATS) and the International Society for Cellular Therapy (ISCT). Cytotherapy.

[63] Mafi P, Hindocha S, Mafi R, Griffin M, Khan W (2011). Adult mesenchymal stem cells and cell surface characterization - a systematic review of the literature. Open Orthop J.

[64] Wagey R, Short B. Mesenchymal stem and progenitor cells: problems, potential and promise. J Stem Cells Res Rev Rep. 2014;1(3):1016.

[65] Wilson A, Hodgson-Garms M, Frith JE, Genever P. Multiplicity of mesenchymal stromal cells: finding the right route to therapy. Frontiers in Immunology. 2019;10. 10.3389/fimmu.2019.01112. 10.3389/fimmu.2019.01112 PMC6535495 31164890

[66] Ioannidis JP (2015). Stealth research: is biomedical innovation happening outside the peer-reviewed literature?. JAMA.

[67] Cristea IA, Cahan EM, Ioannidis JPA (2019). Stealth research: lack of peer-reviewed evidence from healthcare unicorns. Eur J Clin Invest.

[68] Chu CR, Rodeo S, Bhutani N, Goodrich LR, Huard J, Irrgang J, LaPrade RF, Lattermann C, Lu Y, Mandelbaum B, Mao J, McIntyre L, Mishra A, Muschler GF, Piuzzi NS, Potter H, Spindler K, Tokish JM, Tuan R, Zaslav K, Maloney W (2019). Optimizing clinical use of biologics in orthopaedic surgery: consensus recommendations from the 2018 AAOS/NIH U-13 conference. J Am Acad Orthop Surg.

[69] Galleu A, Riffo-Vasquez Y, Trento C, Lomas C, Dolcetti L, Cheung TS, von Bonin M, Barbieri L, Halai K, Ward S, Weng L, Chakraverty R, Lombardi G, Watt FM, Orchard K, Marks DI, Apperley J, Bornhauser M, Walczak H, Bennett C, Dazzi F (2017). Apoptosis in mesenchymal stromal cells induces in vivo recipient-mediated immunomodulation. Sci Transl Med.

[70] Weiss DJ, English K, Krasnodembskaya A, Isaza-Correa JM, Hawthorne IJ, Mahon BP. The necrobiology of mesenchymal stromal cells affects therapeutic efficacy. Frontiers in Immunology. 2019;10. 10.3389/fimmu.2019.01228. 10.3389/fimmu.2019.01228 PMC6557974 31214185

[71] de Witte SFH (2018). immunomodulation by therapeutic mesenchymal stromal cells (MSC) is triggered through phagocytosis of MSC by monocytic cells. Stem Cells.

[72] PRISMA. 2020. http://www.prisma-statement.org/Endorsement/PRISMAEndorsers. Accessed 23 Sept 2020

[73] CONSORT. 2010. http://www.consort-statement.org/. Accessed 23 Oct 2020

[74] Moher D, Hopewell S, Schulz KF, Montori V, Gotzsche PC, Devereaux PJ, Elbourne D, Egger M, Altman DG (2010). CONSORT 2010 explanation and elaboration: updated guidelines for reporting parallel group randomised trials. BMJ.

[75] Gagnier JJ, Boon H, Rochon P, Moher D, Barnes J, Bombardier C, CONSORT Group (2006). Recommendations for reporting randomized controlled trials of herbal interventions: explanation and elaboration. J Clin Epidemiol.

[76] Moher D, Jones A, Lepage L (2001). Use of the CONSORT statement and quality of reports of randomized trials: a comparative before-and-after evaluation. JAMA.

[77] Turner L, Shamseer L, Altman DG, Schulz KF, Moher D (2012). Does use of the CONSORT Statement impact the completeness of reporting of randomised controlled trials published in medical journals? A Cochrane review. Syst Rev.

[78] Johansen M, Thomsen SF (2016). Guidelines for reporting medical research: a critical appraisal. Int Sch Res Notices.

