# Supplementary material for: Characterisation of mesenchymal stromal cells in clinical trial reports: analysis of published descriptors
Source: Stem Cell Res Ther. 2021 Jun 22;12:360. doi: 10.1186/s13287-021-02435-1 (PMC8220718; doi:10.1186/s13287-021-02435-1)
Supplement: Supplementary file 1 — Additional file 1: Table S1. Clinical Trial Summary Information. [file 13287_2021_2435_MOESM1_ESM.doc]

**Title:** **Characterization of mesenchymal stromal cells in clinical trial reports:**

**analysis of published descriptors**

**Authors:** Alison J. Wilson^1^*, Emma Rand^1^, Andrew J. Webster^2^, Paul G. Genever^1^

**Affiliations:**

^1^Department of Biology, University of York, York YO10 5DD, UK.

^2^Science and Technology Studies Unit, Department of Sociology, University of York, York YO10 5DD, UK.

*Corresponding Author. E-mail: [ajw638@york.ac.uk](mailto:ajw638@york.ac.uk)

**SUPPLEMENTARY INFORMATION**

Table S1: Clinical Trial Summary Information

| **Ref** | **Year** | **Clinical trial phase** | **Country** | **Mechanism of action** | **Indication** | **Route** |
| --- | --- | --- | --- | --- | --- | --- |
| **(1)** | 2019 | I/IIa | Canada | multi | Osteoarthritis | Intraarticular |
| **(2)** | 2019 | IIb | Korea | paracrine | Osteoarthritis | Intraarticular |
| **(3)** | 2019 | I | USA | NS** | Tumour homing | Intravenous |
| **(4)** | 2019 | I* | Poland | NS** | Amyotrophic lateral sclerosis | Intrathecal |
| **(5)** | 2018 | I/II | Chile | paracrine | Osteoarthritis | Intraarticular |
| **(6)** | 2018 | II* | China | multi | Cerebral palsy | Intravenous |
| **(7)** | 2018 | I* | USA | immune | Bronchiolitis obliterans syndrome | Intravenous |
| **(8)** | 2018 | I/IIa* | Russia | immune | Haematopoietic stem cell graft failure | Intraosseous |
| **(9)** | 2018 | I | USA | paracrine | Islet graft survival | Local Injection |
| **(10)** | 2018 | I/IIa* | Poland | paracrine | Epilepsy | Intrathecal |
| **(11)** | 2017 | I/IIa* | China | immune | Liver transplant | Intravenous |
| **(12)** | 2017 | I | Denmark | NS** | Ischaemic heart failure | Intracardiac |
| **(13)** | 2017 | I/II | Spain | NS** | Intervertebral disc repair | Local Injection |
| **(14)** | 2017 | II | China | NS** | Aplastic anemia | Intravenous |
| **(15)** | 2017 | I | Australia | paracrine** | Chronic lung allograft dysfunction | Intravenous |
| **(16)** | 2017 | I/IIa* | UK | paracrine | Meniscus repair | Implant |
| **(17)** | 2017 | II | India | paracrine | Critical limb ischaemia | Intramuscular |
| **(18)** | 2017 | I | Brazil | paracrine** | Pulmonary emphysema | Local Injection |
| **(19)** | 2017 | I/II | Korea | paracrine | Osteoarthritis | Intraarticular |
| **(20)** | 2017 | II | Denmark | paracrine | Chronic ischaemic HD | Intracardiac |
| **(21)** | 2017 | I/IIa* | India | paracrine | Stroke | Intravenous |
| **(22)** | 2017 | I/IIa* | China | paracrine | Spinal cord injury | Implant |
| **(23)** | 2017 | I/II | Brazil | NS | Spinal cord injury | Local Injection |
| **(24)** | 2017 | III* | China | paracrine | Spastic cerebral palsy | Intrathecal |
| **(25)** | 2017 | I/II | Chile | paracrine | Heart failure | Intravenous |
| **(26)** | 2017 | I/IIa | Taiwan | NS** | Spinocerebellar ataxia | Intravenous |
| **(27)** | 2017 | II* | Spain | paracrine | Spinal cord injury | Intrathecal |
| **(28)** | 2017 | I/II | USA | NS** | Non-ischaemic dilated cardiomyopathy | Intracardiac |
| **(29)** | 2016 | I/IIa | Spain | NS** | Crohn's disease fistula | Local Injection |
| **(30)** | 2016 | IIa* | Indonesia | NS** | Bone non-union | Implant |
| **(31)** | 2016 | I | Multi | paracrine | Osteoarthritis | Intraarticular |
| **(32)** | 2016 | I/II | Japan | differentiation | Periodontitis | Implant |
| **(33)** | 2016 | I/IIa* | Turkey | immune** | Graft-vs-host disease | Intravenous |
| **(34)** | 2016 | I/II | Spain | paracrine | Osteoarthritis | Intrathecal |
| **(35)** | 2016 | I | Korea | NS** | Spinal cord injury | Intrathecal |
| **(36)** | 2016 | III | multi | immune | Crohn's disease fistula | Local Injection |
| **(37)** | 2016 | II* | Korea | multi | Crohn's disease fistula | Local Injection |
| **(38)** | 2016 | I | Israel | NS | Amyotrophic lateral sclerosis | Intrathecal |
| **(39)** | 2016 | I | Pakistan | paracrine | Spinal cord injury | Intrathecal |
| **(40)** | 2016 | I/IIa* | USA | NS | Amyotrophic lateral sclerosis | Intrathecal |
| **(41)** | 2016 | I/II | USA | NS | Chronic ischaemic stroke | Local Injection |
| **(42)** | 2016 | I/II | Spain | NS | Chronic complete paraplegia | Intrathecal |
| **(43)** | 2015 | I | Spain | immune** | Osteoarthritis | Intraarticular |
| **(44)** | 2015 | I | Korea | NS | Amyotrophic lateral sclerosis | Intrathecal |
| **(45)** | 2015 | I/II* | Poland | NS** | Acute myocardial infarction | Intracoronary |
| **(46)** | 2015 | IIb | India | multi** | Acute myocardial infarction | Intravenous |
| **(47)** | 2015 | II* | China | paracrine | Acute myocardial infarction | Intracoronary |
| **(48)** | 2015 | I/IIa | Denmark | immune | Ischaemic heart failure | Intracardiac |
| **(49)** | 2015 | II | USA | paracrine | Non-ischaemic and ischaemic heart failure | Intracardiac |
| **(50)** | 2015 | II* | China | NS** | Spastic cerebral palsy | Intrathecal |
| **(51)** | 2014 | II | China | paracrine | Ankylosing spondylitis | Intravenous |
| **(52)** | 2014 | I/IIa* | China | immune | Poor graft function | Intravenous |
| **(53)** | 2014 | II* | China | multi | Spinal cord injury | Local Injection |
| **(54)** | 2014 | II* | Korea | NS | Osteoarthritis | Intraarticular |
| **(55)** | 2014 | IIa | USA | multi | Coronary artery bypass graft | Intracardiac |
| **(56)** | 2014 | II* | Korea | paracrine | Amyotrophic lateral sclerosis | Intrathecal |
| **(57)** | 2014 | II* | Spain | immune | Multiple sclerosis | Intravenous |
| **(58)** | 2014 | I | Brazil | NS | Spinal cord injury | Local Injection |
| **(59)** | 2014 | I/IIa* | China | paracrine | Ischaemic stroke | Intravenous |
| **(60)** | 2014 | II* | Vietnam | paracrine | Osteoarthritis | Local Injection |
| **(61)** | 2014 | I/II | USA | paracrine | Ischaemic cardiomyopathy | Intracardiac |
| **(62)** | 2013 | II* | China | NS | Spinal cord injury | Local Injection |
| **(63)** | 2013 | I/II | Spain | multi** | Osteoarthritis | Intraarticular |
| **(64)** | 2013 | I/IIa* | Taiwan | immune | Engraftment following cord blood transplant | Intravenous |
| **(65)** | 2013 | IIa* | Italy | multi | Systemic sclerosis | Subcutaneous |
| **(66)** | 2013 | I | Korea | multi | Perianal fistula (Crohn's) | Local Injection |
| **(67)** | 2013 | I/IIa | Spain | multi | Perianal fistula (Crohn's) | Local Injection |
| **(68)** | 2013 | II* | China | NS** | Acute myocardial infarction | Intracoronary |
| **(69)** | 2013 | IIa* | Denmark | NS** | Refractory angina | Intracardiac |
| **(70)** | 2013 | I/IIa* | China | NS** | Hereditary spinocerebellar ataxia | Intrathecal |
| **(71)** | 2013 | I/IIa* | Netherlands | NS | Acute myocardial infarction | Intracardiac |
| **(72)** | 2013 | Ib | Greece | NS | Idiopathic pulmonary fibrosis | Intravenous |
| **(73)** | 2013 | II* | China | multi | Traumatic brain injury | Intrathecal |
| **(74)** | 2013 | II | USA | paracrine | Chronic pulmonary obstructive disease | Intravenous |
| **(75)** | 2012 | II | Russia | immune | Graft-vs-host disease | Intravenous |
| **(76)** | 2012 | I/II | Netherlands | NS | Myocardial infarction | Intracoronary |
| **(77)** | 2012 | I/II | Iran | multi | Spinal cord injury | Intrathecal |
| **(78)** | 2012 | I/IIa | USA | NS | Ischaemic cardiomyopathy | Intracardiac |
| **(79)** | 2011 | I* | Denmark | multi** | Angina | Intracardiac |
| **(80)** | 2011 | I* | Spain | immune | Intra-vertebral disc repair | Local Injection |
| **(81)** | 2011 | I* | Taiwan | NS | Graft-vs-host disease | Intravenous |
| **(82)** | 2011 | I/IIa | Japan | paracrine** | Stroke | Intravenous |
| **(83)** | 2010 | II* | Egypt | NS | Spinal cord injury | Intrathecal |
| **(84)** | 2010 | II* | Korea | NS | Ischaemic stroke | Intravenous |

*Phase assigned during review

**MOA assigned during review

1. Chahal J, Gómez-Aristizábal A, Shestopaloff K, Bhatt S, Chaboureau A, Fazio A, et al. Bone Marrow Mesenchymal Stromal Cell Treatment in Patients with Osteoarthritis Results in Overall Improvement in Pain and Symptoms and Reduces Synovial Inflammation. STEM CELLS Translational Medicine. 2019;8(8):746-757.

2. Lee W-S, Kim HJ, Kim K-I, Kim GB, Jin W. Intra-Articular Injection of Autologous Adipose Tissue-Derived Mesenchymal Stem Cells for the Treatment of Knee Osteoarthritis: A Phase IIb, Randomized, Placebo-Controlled Clinical Trial. STEM CELLS Translational Medicine. 2019;8(6):504-111.

3. Schweizer MT, Wang H, Bivalacqua TJ, Partin AW, Lim SJ, Chapman C, et al. A Phase I Study to Assess the Safety and Cancer-Homing Ability of Allogeneic Bone Marrow-Derived Mesenchymal Stem Cells in Men with Localized Prostate Cancer. STEM CELLS Translational Medicine. 2019;8(5):441-449.

4. Barczewska M, Grudniak M, Maksymowicz S, Siwek T, Ołdak T, Jezierska-Woźniak K, et al. Safety of intrathecal injection of Wharton’s jelly-derived mesenchymal stem cells in amyotrophic lateral sclerosis therapy. Neural Regen Res. 2019;14(2):313-318.

5. Matas J, Orrego M, Amenabar D, Infante C, Tapia-Limonchi R, Cadiz MI, et al. Umbilical Cord-Derived Mesenchymal Stromal Cells (MSCs) for Knee Osteoarthritis: Repeated MSC Dosing Is Superior to a Single MSC Dose and to Hyaluronic Acid in a Controlled Randomized Phase I/II Trial. STEM CELLS Translational Medicine. 2019;8(3):215-224.

6. Huang L, Zhang C, Gu J, Wu W, Shen Z, Zhou X, et al. A Randomized, Placebo-Controlled Trial of Human Umbilical Cord Blood Mesenchymal Stem Cell Infusion for Children With Cerebral Palsy. Cell Transplant. 2018;27(2):325-334.

7. Keller CA, Gonwa TA, Hodge DO, Hei DJ, Centanni JM, Zubair AC. Feasibility, Safety, and Tolerance of Mesenchymal Stem Cell Therapy for Obstructive Chronic Lung Allograft Dysfunction. Stem Cells Transl Med. 2018;7(2):161-167.

8. Petinati N, Drize N, Sats N, Risinskaya N, Sudarikov A, Drokov M, et al. Recovery of Donor Hematopoiesis after Graft Failure and Second Hematopoietic Stem Cell Transplantation with Intraosseous Administration of Mesenchymal Stromal Cells. Stem Cells Int. 2018;2018.

9. Wang H, Strange C, Nietert PJ, Wang J, Turnbull TL, Cloud C, et al. Autologous Mesenchymal Stem Cell and Islet Cotransplantation: Safety and Efficacy. Stem Cells Transl Med. 2018;7(1):11-19.

10. Milczarek O, Jarocha D, Starowicz–Filip A, Kwiatkowski S, Badyra B, Majka M. Multiple Autologous Bone Marrow-Derived CD271+ Mesenchymal Stem Cell Transplantation Overcomes Drug-Resistant Epilepsy in Children. STEM CELLS Translational Medicine. 2018;7(1):20-33.

11. Shi M, Liu Z, Wang Y, Xu R, Sun Y, Zhang M, et al. A Pilot Study of Mesenchymal Stem Cell Therapy for Acute Liver Allograft Rejection. Stem Cells Transl Med. 2017;6(12):2053-2061.

12. Kastrup J, Haack‐Sørensen M, Juhl M, Harary Søndergaard R, Follin B, Drozd Lund L, et al. Cryopreserved Off‐the‐Shelf Allogeneic Adipose‐Derived Stromal Cells for Therapy in Patients with Ischemic Heart Disease and Heart Failure—A Safety Study. Stem Cells Transl Med. 2017;6(11):1963-1671.

13. Noriega DC, Ardura F, Hernández-Ramajo R, Martín-Ferrero MÁ, Sánchez-Lite I, Toribio B, et al. Intervertebral Disc Repair by Allogeneic Mesenchymal Bone Marrow Cells: A Randomized Controlled Trial. Transplantation. 2017;101(8):1945-1951.

14. Pang Y, Xiao H-W, Zhang H, Liu Z-H, Li L, Gao Y, et al. Allogeneic Bone Marrow-Derived Mesenchymal Stromal Cells Expanded In Vitro for Treatment of Aplastic Anemia: A Multicenter Phase II Trial. STEM CELLS Translational Medicine. 2017;6(7):1569-1575.

15. Chambers DC, Enever D, Lawrence S, Sturm MJ, Herrmann R, Yerkovich S, et al. Mesenchymal Stromal Cell Therapy for Chronic Lung Allograft Dysfunction: Results of a First-in-Man Study. STEM CELLS Translational Medicine. 2017;6(4):1152-1157.

16. Whitehouse MR, Howells NR, Parry MC, Austin E, Kafienah W, Brady K, et al. Repair of Torn Avascular Meniscal Cartilage Using Undifferentiated Autologous Mesenchymal Stem Cells: From In Vitro Optimization to a First-in-Human Study. STEM CELLS Translational Medicine. 2017;6(4):1237-1248.

17. Gupta PK, Krishna M, Chullikana A, Desai S, Murugesan R, Dutta S, et al. Administration of Adult Human Bone Marrow-Derived, Cultured, Pooled, Allogeneic Mesenchymal Stromal Cells in Critical Limb Ischemia Due to Buerger’s Disease: Phase II Study Report Suggests Clinical Efficacy. STEM CELLS Translational Medicine. 2017;6(3):689-699.

18. de Oliveira HG, Cruz FF, Antunes MA, de Macedo Neto AV, Oliveira GA, Svartman FM, et al. Combined Bone Marrow-Derived Mesenchymal Stromal Cell Therapy and One-Way Endobronchial Valve Placement in Patients with Pulmonary Emphysema: A Phase I Clinical Trial. STEM CELLS Translational Medicine. 2017;6(3):962-969.

19. Park Y, Ha C, Lee C, Yoon YC. Cartilage Regeneration in Osteoarthritic Patients by a Composite of Allogeneic Umbilical Cord Blood‐Derived Mesenchymal Stem Cells and Hyaluronate Hydrogel: Results from a Clinical Trial for Safety and Proof‐of‐Concept with 7 Years of Extended Follow‐Up. Stem Cells Transl Med. 2017;6(2):613-621.

20. Qayyum AA, Mathiasen AB, Mygind ND, Kühl JT, Jørgensen E, Helqvist S, et al. Adipose-Derived Stromal Cells for Treatment of Patients with Chronic Ischemic Heart Disease (MyStromalCell Trial): A Randomized Placebo-Controlled Study. Stem Cells Int. 2017;2017.

21. Bhasin A, Kumaran SS, Bhatia R, Mohanty S, Srivastava MVP. Safety and Feasibility of Autologous Mesenchymal Stem Cell Transplantation in Chronic Stroke in Indian patients. A four-year follow up. J Stem Cells Regen Med. 2017;13(1):14-19.

22. Zhao Y, Tang F, Xiao Z, Han G, Wang N, Yin N, et al. Clinical Study of NeuroRegen Scaffold Combined With Human Mesenchymal Stem Cells for the Repair of Chronic Complete Spinal Cord Injury. Cell transplantation. 2017;26(5):891-900.

23. Larocca TF, Macêdo CT, Souza BSdF, Andrade-Souza YM, Villarreal CF, Matos AC, et al. Image-guided percutaneous intralesional administration of mesenchymal stromal cells in subjects with chronic complete spinal cord injury: a pilot study. Cytotherapy. 2017;19(10):1189-1196.

24. Liu X, Fu X, Dai G, Wang X, Zhang Z, Cheng H, et al. Comparative analysis of curative effect of bone marrow mesenchymal stem cell and bone marrow mononuclear cell transplantation for spastic cerebral palsy. J Transl Med. 2017;15.

25. Bartolucci J, Verdugo FJ, González PL, Larrea RE, Abarzua E, Goset C, et al. Safety and Efficacy of the Intravenous Infusion of Umbilical Cord Mesenchymal Stem Cells in Patients With Heart Failure: A Phase 1/2 Randomized Controlled Trial (RIMECARD Trial [Randomized Clinical Trial of Intravenous Infusion Umbilical Cord Mesenchymal Stem Cells on Cardiopathy]). Circ Res. 2017;121(10):1192-1204.

26. Tsai YA, Liu RS, Lirng JF, Yang BH, Chang CH, Wang YC, et al. Treatment of Spinocerebellar Ataxia with Mesenchymal Stem Cells: A Phase I/IIa Clinical Study. Cell Transplant. 2017;26(3):503-512.

27. Vaquero J, Zurita M, Rico MA, Bonilla C, Aguayo C, Fernández C, et al. Repeated subarachnoid administrations of autologous mesenchymal stromal cells supported in autologous plasma improve quality of life in patients suffering incomplete spinal cord injury. Cytotherapy. 2017;19(3):349-359.

28. Hare JM, DiFede DL, Castellanos AM, Florea V, Landin AM, El-Khorazaty J, et al. Randomized Comparison of Allogeneic Vs. Autologous Mesenchymal Stem Cells for Non-lschemic Dilated Cardiomyopathy: POSEIDON-DCM Trial. J Am Coll Cardiol. 2017;69(5):526-337.

29. García-Arranz M, Herreros MD, González-Gómez C, de la Quintana P, Guadalajara H, Georgiev-Hristov T, et al. Treatment of Crohn's-Related Rectovaginal Fistula With Allogeneic Expanded-Adipose Derived Stem Cells: A Phase I–IIa Clinical Trial. STEM CELLS Translational Medicine. 2016;5(11):1441-1446.

30. Ismail H, Phedy P, Kholinne E, Djaja YP, Kusnadi Y, Merlina M, et al. Mesenchymal stem cell implantation in atrophic nonunion of the long bones: A translational study. Bone Joint Res. 2016;5(7):287-293.

31. Pers Y-M, Rackwitz L, Ferreira R, Pullig O, Delfour C, Barry F, et al. Adipose Mesenchymal Stromal Cell-Based Therapy for Severe Osteoarthritis of the Knee: A Phase I Dose-Escalation Trial. STEM CELLS Translational Medicine. 2016;5(7):847-856.

32. Baba S, Yamada Y, Komuro A, Yotsui Y, Umeda M, Shimuzutani K, et al. Phase I/II Trial of Autologous Bone Marrow Stem Cell Transplantation with a Three-Dimensional Woven-Fabric Scaffold for Periodontitis. Stem Cells Int. 2016;2016:6205910.

33. Erbey F, Atay D, Akcay A, Ovali E, Ozturk G. Mesenchymal Stem Cell Treatment for Steroid Refractory Graft-versus-Host Disease in Children: A Pilot and First Study from Turkey. Stem Cells Int. 2016;2016:1641402.

34. Lamo-Espinosa JM, Mora G, Blanco JF, Granero-Moltó F, Nuñez-Córdoba JM, Sánchez-Echenique C, et al. Intra-articular injection of two different doses of autologous bone marrow mesenchymal stem cells versus hyaluronic acid in the treatment of knee osteoarthritis: multicenter randomized controlled clinical trial (phase I/II). Journal of Translational Medicine. 2016;14(1):246.

35. Hur JW, Cho TH, Park DH, Lee JB, Park JY, Chung YG. Intrathecal transplantation of autologous adipose-derived mesenchymal stem cells for treating spinal cord injury: A human trial. J Spinal Cord Med. 2016;39(6):655-664.

36. Panés J, García-Olmo D, Van Assche G, Colombel JF, Reinisch W, Baumgart DC, et al. Expanded allogeneic adipose-derived mesenchymal stem cells (Cx601) for complex perianal fistulas in Crohn's disease: a phase 3 randomised, double-blind controlled trial. Lancet. 2016;388(10051):1281-1290.

37. Park KJ, Ryoo SB, Kim JS, Kim TI, Baik SH, Kim HJ, et al. Allogeneic adipose-derived stem cells for the treatment of perianal fistula in Crohn's disease: a pilot clinical trial. Colorectal Dis. 2016;18(5):468-476.

38. Petrou P, Gothelf Y, Argov Z, Gotkine M, Levy YS, Kassis I, et al. Safety and Clinical Effects of Mesenchymal Stem Cells Secreting Neurotrophic Factor Transplantation in Patients With Amyotrophic Lateral Sclerosis: Results of Phase 1/2 and 2a Clinical Trials. JAMA Neurol. 2016;73(3):337-344.

39. Satti HS, Waheed A, Ahmed P, Ahmed K, Akram Z, Aziz T, et al. Autologous mesenchymal stromal cell transplantation for spinal cord injury: A Phase I pilot study. Cytotherapy. 2016;18(4):518-222.

40. Staff NP, Madigan NN, Morris J, Jentoft M, Sorenson EJ, Butler G, et al. Safety of intrathecal autologous adipose-derived mesenchymal stromal cells in patients with ALS. Neurology. 2016;87(21):2230-2234.

41. Steinberg GK, Kondziolka D, Wechsler LR, Lunsford LD, Coburn ML, Billigen JB, et al. Clinical Outcomes of Transplanted Modified Bone Marrow–Derived Mesenchymal Stem Cells in Stroke: A Phase 1/2a Study. Stroke. 2016;47(7):1817-1824.

42. Vaquero J, Zurita M, Rico MA, Bonilla C, Aguayo C, Montilla J, et al. An approach to personalized cell therapy in chronic complete paraplegia: The Puerta de Hierro phase I/II clinical trial. Cytotherapy. 2016;18(8):1025-1036.

43. Vega A, Martín-Ferrero MA, Del Canto F, Alberca M, García V, Munar A, et al. Treatment of Knee Osteoarthritis With Allogeneic Bone Marrow Mesenchymal Stem Cells: A Randomized Controlled Trial. Transplantation. 2015;99(8):1681-1690.

44. Oh K-W, Moon C, Kim HY, Oh S-i, Park J, Lee JH, et al. Phase I Trial of Repeated Intrathecal Autologous Bone Marrow-Derived Mesenchymal Stromal Cells in Amyotrophic Lateral Sclerosis. STEM CELLS Translational Medicine. 2015;4(6):590-597.

45. Musialek P, Mazurek A, Jarocha D, Tekieli L, Szot W, Kostkiewicz M, et al. Myocardial regeneration strategy using Wharton's jelly mesenchymal stem cells as an off-the-shelf ‘unlimited’ therapeutic agent: results from the Acute Myocardial Infarction First-in-Man Study. Postepy Kardiol Interwencyjnej. 2015;11(2):100-107.

46. Chullikana A, Majumdar AS, Gottipamula S, Krishnamurthy S, Kumar AS, Prakash VS, et al. Randomized, double-blind, phase I/II study of intravenous allogeneic mesenchymal stromal cells in acute myocardial infarction. Cytotherapy. 2015;17(3):250-261.

47. Gao LR, Chen Y, Zhang NK, Yang XL, Liu HL, Wang ZG, et al. Intracoronary infusion of Wharton’s jelly-derived mesenchymal stem cells in acute myocardial infarction: double-blind, randomized controlled trial. BMC Med. 2015;13.

48. Mathiasen AB, Qayyum AA, Jørgensen E, Helqvist S, Fischer-Nielsen A, Kofoed KF, et al. Bone marrow-derived mesenchymal stromal cell treatment in patients with severe ischaemic heart failure: a randomized placebo-controlled trial (MSC-HF trial). Eur Heart J. 2015;36(27):1744-1753.

49. Perin EC, Borow KM, Silva GV, DeMaria AN, Marroquin OC, Huang PP, et al. A Phase II Dose-Escalation Study of Allogeneic Mesenchymal Precursor Cells in Patients With Ischemic or Nonischemic Heart Failure. Circ Res. 2015;117(6):576-584.

50. Wang X, Hu H, Hua R, Yang J, Zheng P, Niu X, et al. Effect of umbilical cord mesenchymal stromal cells on motor functions of identical twins with cerebral palsy: pilot study on the correlation of efficacy and hereditary factors. Cytotherapy. 2015;17(2):224-231.

51. Wang P, Li Y, Huang L, Yang J, Yang R, Deng W, et al. Effects and safety of allogenic mesenchymal stem cell intravenous infusion in active ankylosing spondylitis patients who failed NSAIDs: a 20-week clinical trial. Cell Transplant. 2014;23(10):1293-1303.

52. Liu X, Wu M, Peng Y, Chen X, Sun J, Huang F, et al. Improvement in poor graft function after allogeneic hematopoietic stem cell transplantation upon administration of mesenchymal stem cells from third-party donors: a pilot prospective study. Cell Transplant. 2014;23(9):1087-1098.

53. Cheng H, Liu X, Hua R, Dai G, Wang X, Gao J, et al. Clinical observation of umbilical cord mesenchymal stem cell transplantation in treatment for sequelae of thoracolumbar spinal cord injury. J Transl Med. 2014;12.

54. Jo CH, Lee YG, Shin WH, Kim H, Chai JW, Jeong EC, et al. Intra-Articular Injection of Mesenchymal Stem Cells for the Treatment of Osteoarthritis of the Knee: A Proof-of-Concept Clinical Trial. STEM CELLS. 2014;32(5):1254-1266.

55. Karantalis V, DiFede DL, Gerstenblith G, Pham S, Symes J, Zambrano JP, et al. Autologous Mesenchymal Stem Cells Produce Concordant Improvements in Regional Function, Tissue Perfusion and Fibrotic Burden when Administered to Patients Undergoing Coronary Artery Bypass Grafting – The PROMETHEUS Trial. Circ Res. 2014;114(8):1302-1310.

56. Kim HY, Kim H, Oh KW, Oh SI, Koh SH, Baik W, et al. Biological markers of mesenchymal stromal cells as predictors of response to autologous stem cell transplantation in patients with amyotrophic lateral sclerosis: an investigator-initiated trial and in vivo study. Stem Cells. 2014;32(10):2724-2731.

57. Llufriu S, Sepúlveda M, Blanco Y, Marín P, Moreno B, Berenguer J, et al. Randomized Placebo-Controlled Phase II Trial of Autologous Mesenchymal Stem Cells in Multiple Sclerosis. PLoS One. 2014;9(12).

58. Mendonça MVP, Larocca TF, de Freitas Souza BS, Villarreal CF, Silva LFM, Matos AC, et al. Safety and neurological assessments after autologous transplantation of bone marrow mesenchymal stem cells in subjects with chronic spinal cord injury. Stem Cell Res Ther. 2014;5(6).

59. Qiao LY, Huang FJ, Zhao M, Xie JH, Shi J, Wang J, et al. A two-year follow-up study of cotransplantation with neural stem/progenitor cells and mesenchymal stromal cells in ischemic stroke patients. Cell Transplant. 2014;23 Suppl 1:S65-72.

60. Bui K, Duong T, Nguyen N, Nguyen T, Le V, Mai V, et al. Symptomatic knee osteoarthritis treatment using autologous adipose derived stem cells and platelet-rich plasma: a clinical study. *Biomedical Research and Therapy*. 2014;1 (01):02-08.

61. Heldman AW, DiFede DL, Fishman JE, Zambrano JP, Trachtenberg BH, Karantalis V, et al. Transendocardial Mesenchymal Stem Cells and Mononuclear Bone Marrow Cells for Ischemic Cardiomyopathy: The TAC-HFT Randomized Trial. JAMA. 2014;311(1):62-73.

62. Dai G, Liu X, Zhang Z, Yang Z, Dai Y, Xu R. Transplantation of autologous bone marrow mesenchymal stem cells in the treatment of complete and chronic cervical spinal cord injury. Brain Research. 2013;1533:73-79.

63. Orozco L, Munar A, Soler R, Alberca M, Soler F, Huguet M, et al. Treatment of Knee Osteoarthritis With Autologous Mesenchymal Stem Cells: A Pilot Study. Transplantation. 2013;95(12):1535-1541.

64. Wu K-H, Sheu J-N, Wu H-P, Tsai C, Sieber M, Peng C-T, et al. Cotransplantation of Umbilical Cord–Derived Mesenchymal Stem Cells Promote Hematopoietic Engraftment in Cord Blood Transplantation: A Pilot Study. Transplantation. 2013;95(5):773-7777.

65. Scuderi N, Ceccarelli S, Onesti MG, Fioramonti P, Guidi C, Romano F, et al. Human adipose-derived stromal cells for cell-based therapies in the treatment of systemic sclerosis. Cell Transplant. 2013;22(5):779-795.

66. Cho YB, Lee WY, Park KJ, Kim M, Yoo HW, Yu CS. Autologous adipose tissue-derived stem cells for the treatment of Crohn's fistula: a phase I clinical study. Cell Transplant. 2013;22(2):279-285.

67. de la Portilla F, Alba F, García-Olmo D, Herrerías JM, González FX, Galindo A. Expanded allogeneic adipose-derived stem cells (eASCs) for the treatment of complex perianal fistula in Crohn’s disease: results from a multicenter phase I/IIa clinical trial. International Journal of Colorectal Disease. 2013;28(3):313-323.

68. Gao LR, Pei XT, Ding QA, Chen Y, Zhang NK, Chen HY, et al. A critical challenge: dosage-related efficacy and acute complication intracoronary injection of autologous bone marrow mesenchymal stem cells in acute myocardial infarction. Int J Cardiol. 2013;168(4):3191-3199.

69. Haack-Sørensen M, Friis T, Mathiasen AB, Jørgensen E, Hansen L, Dickmeiss E, et al. Direct intramyocardial mesenchymal stromal cell injections in patients with severe refractory angina: one-year follow-up. Cell Transplant. 2013;22(3):521-528.

70. Jia-Li J, Zhuo L, Zhen-Juan L, De-Ning G, Chong W, Zhi-Bin C, et al. Safety and Efficacy of Umbilical Cord Mesenchymal Stem Cell Therapy in Hereditary Spinocerebellar Ataxia. Current Neurovascular Research. 2013;10(1):11-20.

71. Rodrigo SF, van Ramshorst J, Hoogslag GE, Boden H, Velders MA, Cannegieter SC, et al. Intramyocardial Injection of Autologous Bone Marrow-Derived Ex Vivo Expanded Mesenchymal Stem Cells in Acute Myocardial Infarction Patients is Feasible and Safe up to 5 Years of Follow-up. J Cardiovasc Transl Res. 2013;6(5):816-825.

72. Tzouvelekis A, Paspaliaris V, Koliakos G, Ntolios P, Bouros E, Oikonomou A, et al. A prospective, non-randomized, no placebo-controlled, phase Ib clinical trial to study the safety of the adipose derived stromal cells-stromal vascular fraction in idiopathic pulmonary fibrosis. J Transl Med. 2013;11:171.

73. Wang S, Cheng H, Dai G, Wang X, Hua R, Liu X, et al. Umbilical cord mesenchymal stem cell transplantation significantly improves neurological function in patients with sequelae of traumatic brain injury. Brain Research. 2013;1532:76-84.

74. Weiss DJ, Casaburi R, Flannery R, LeRoux-Williams M, Tashkin DP. A Placebo-Controlled, Randomized Trial of Mesenchymal Stem Cells in COPD. Chest. 2013;143(6):1590-1598.

75. Kuzmina LA, Petinati NA, Parovichnikova EN, Lubimova LS, Gribanova EO, Gaponova TV, et al. Multipotent Mesenchymal Stromal Cells for the Prophylaxis of Acute Graft-versus-Host Disease—A Phase II Study. Stem Cells Int. 2012;2012.

76. Houtgraaf JH, den Dekker WK, van Dalen BM, Springeling T, de Jong R, van Geuns RJ, et al. First Experience in Humans Using Adipose Tissue–Derived Regenerative Cells in the Treatment of Patients With ST-Segment Elevation Myocardial Infarction. Journal of the American College of Cardiology. 2012;59(5):539-540.

77. Karamouzian S, Nematollahi-Mahani SN, Nakhaee N, Eskandary H. Clinical safety and primary efficacy of bone marrow mesenchymal cell transplantation in subacute spinal cord injured patients. Clinical Neurology and Neurosurgery. 2012;114(7):935-939.

78. Hare JM, Fishman JE, Gerstenblith G, DiFede Velazquez DL, Zambrano JP, Suncion VY, et al. Comparison of Allogeneic vs Autologous Bone Marrow–Derived Mesenchymal Stem Cells Delivered by Transendocardial Injection in Patients With Ischemic Cardiomyopathy: The POSEIDON Randomized Trial. JAMA. 2012;308(22):2369-2379.

79. Friis T, Haack-Sørensen M, Mathiasen AB, Ripa RS, Kristoffersen US, Jørgensen E, et al. Mesenchymal stromal cell derived endothelial progenitor treatment in patients with refractory angina. Scand Cardiovasc J. 2011;45(3):161-168.

80. Orozco L, Soler R, Morera C, Alberca M, Sánchez A, García-Sancho J. Intervertebral Disc Repair by Autologous Mesenchymal Bone Marrow Cells: A Pilot Study. Transplantation. 2011;92(7):822-828.

81. Wu K-H, Chan C-K, Tsai C, Chang Y-H, Sieber M, Chiu T-H, et al. Effective Treatment of Severe Steroid-Resistant Acute Graft-Versus-Host Disease With Umbilical Cord-Derived Mesenchymal Stem Cells. Transplantation. 2011;91(12):1412-1416.

82. Honmou O, Houkin K, Matsunaga T, Niitsu Y, Ishiai S, Onodera R, et al. Intravenous administration of auto serum-expanded autologous mesenchymal stem cells in stroke. Brain. 2011;134(6):1790-1807.

83. Kishk NA, Gabr H, Hamdy S, Afifi L, Abokresha N, Mahmoud H, et al. Case control series of intrathecal autologous bone marrow mesenchymal stem cell therapy for chronic spinal cord injury. Neurorehabil Neural Repair. 2010;24(8):702-708.

84. Lee JS, Hong JM, Moon GJ, Lee PH, Ahn YH, Bang OY. A Long-Term Follow-Up Study of Intravenous Autologous Mesenchymal Stem Cell Transplantation in Patients With Ischemic Stroke. STEM CELLS. 2010;28(6):1099-1106.
